# Using Approximate Bayesian Computation to Calibrate the Model Parameters Characterizing the Autoregulatory Behavior of Microvessels

**DOI:** 10.1002/cnm.70023

**Published:** 2025-03-17

**Authors:** Ali Daher

**Affiliations:** ^1^ Institute of Biomedical Engineering, Department of Engineering Science University of Oxford Oxford UK

**Keywords:** approximate Bayesian computation, autoregulation, compliance feedback model, microvessels, parameter calibration

## Abstract

This study aims to leverage available experimental data on the myogenic and endothelial responses of the microvessels to calibrate the parameters and refine the functional form of the compliance feedback model. The experimental data used in this study trace the changes in the vessel calibre of individual arteriolar vessels in response to changes in the intraluminal pressure and/or the pressure gradient, which correspond to the myogenic and endothelial mechanisms, respectively. The compliance feedback model was previously developed to characterize the elastic and autoregulatory behavior of microvessels. We devise and employ a two‐stage sequential Monte Carlo (MC) approximate Bayesian computation (ABC) scheme to obtain the posterior distribution of the model's parameters, such that the final parameter space distribution integrates information from any prior knowledge of the parameters, the model dynamics, and the available experimental data. Furthermore, the calibration scheme provides key insights into the underlying mechanistic features of the dynamical system; namely, the ABC scheme reveals that there is a marked difference in the time constants between the myogenic‐induced dilation and constriction. Overall, upon parameter calibration, the computationally low‐cost compliance feedback model achieves very good agreement with the experimental measurements, despite limited data availability, demonstrating that the model provides a simple, compact, yet robust and physiologically grounded characterization of the autoregulatory response, all of which are essential attributes to increase the translatability of hemodynamic models into the clinical environment for future clinical applications.

## Graphical abstract

We use experimental data on the myogenic and endothelial responses of individual microvessels to calibrate the compliance feedback model's parameters. A two‐stage sequential Monte Carlo Approximate Bayesian Computation scheme is employed to derive the parameters' posterior distributions, which integrates prior knowledge, model dynamics, and experimental data. This calibration highlights differences in time constants between myogenic‐induced dilation and constriction. The refined model provides a robust, physiologically grounded characterization of autoregulatory response, increasing its potential for clinical application despite limited data.

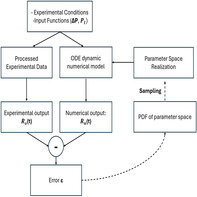

## 1 Introduction

For physiological models to be clinically useful, they need to be grounded in physiological principles and informed by empirical data. In the existing models related to dynamic autoregulation and blood flow regulation within vascular networks, especially in the microvasculature, there has been a notable lack of rigorous efforts to estimate model parameters or to identify which parameters significantly impact the models. Given the very limited data available on the autoregulatory behavior of vessels and networks, it can be challenging to determine the parameter values for models that include relevant laws describing the elastic and autoregulatory behavior of the arteriolar vessels [1]. We have recently developed network models of blood flow control [2, 3, 4] that utilize a simple, computationally efficient compliance feedback model, based on the compartmental models of Ursino and colleagues [5, 6], that captures the interactions between the different blood flow control mechanisms, as well as their effect on the vessel calibre of the individual autoregulating vessels. The use of such simplified models to characterize the autoregulatory behavior of the microvessels can help facilitate parameter assignment from the limited data available and circumvent the significant mathematical intricacy that would otherwise hinder the parameter calibration process. This work aims to utilize available experimental data on the vessels' elastic and autoregulatory behavior, specifically the myogenic and endothelial responses, to calibrate the parameters and functional form of the compliance feedback model that characterizes the changes in the vessel compliance in response to the autoregulatory mechanisms. Robust calibration of the model parameters can help transition models from the mere conceptual to the applicable realm, enabling the configuration of reliable in silico vascular testing beds. These in silico platforms can, in turn, help elucidate and provide insights into mechanistic pathways that are otherwise difficult or impossible to solely examine through in vivo methods. In addition, they can act as virtual safe, low‐cost testing beds [7, 8] that offer preliminary assessments for the different outcomes before carrying out any real‐life clinical interventions or treatment plans, especially those that carry significant risks.

### 1.1 Structural Identifiability

When attempting to calibrate the unknown parameter values of a dynamic model, an important consideration is whether these values can be uniquely determined from observations or experimental measurements. This concept is referred to as structural identifiability. If a model is structurally non‐identifiable, then multiple combinations of parameter values (p∈P) can result in similar model predictions, which can lead to artefacts in the model calibration and hence to errors in the subsequent model predictions [9]. Following a structural identifiability analysis of a model, the free model parameters can be classified into one of the three categories [10]:

1. Structurally globally (or uniquely) identifiable: across the entire parameter space, a particular set of parameters (p∈P) can uniquely explain the observed data, such that for almost any $p^{*}\in P$

(1) $$\sum \left(p\right)=\sum \left(p^{*}\right)\Rightarrow p_{i}=p_{i}^{*}$$

2 Structurally locally identifiable: if a unique parameter realization can explain the observed data, but only within a restricted region of the parameter space $V\left(p^{*}\right)$ such that

(2) $$p\in V\left(p^{*}\right)\;\text{and}\sum \left(p\right)=\sum \left(p^{*}\right)\Rightarrow p_{i}=p_{i}^{*}$$

3 Structurally non‐identifiable: There is no region $V\left(p^{*}\right)$ in the parameter space in which a particular parameter space realization can uniquely explain the data

The structural identifiability of a model is an intrinsic property of the model's structure and dynamics, the output (observations), and any input (control) functions [11]; hence, it can be determined prior to any fitting with observed data. Structural identifiability can, in theory, identify if any parameters are redundant or unidentifiable due, for instance, to model symmetries, parameter redundancies, or indistinguishable effects. A related but distinct concept is that of practical identifiability, which is assessed after utilizing the experimental data to calibrate the parameters and is intimately linked to the quality and information in the experimental data [10]. While structural non‐identifiability is usually caused by over parameterization of the model, practical non‐identifiability is due to the lack of sufficient information in the available data or due to experimental noise, both of which hinder meaningful recovery of the parameter values. Intuitively, structural identifiability is a prerequisite for practical identifiability.

### 1.2 Experimental Data

**FIGURE 1:**
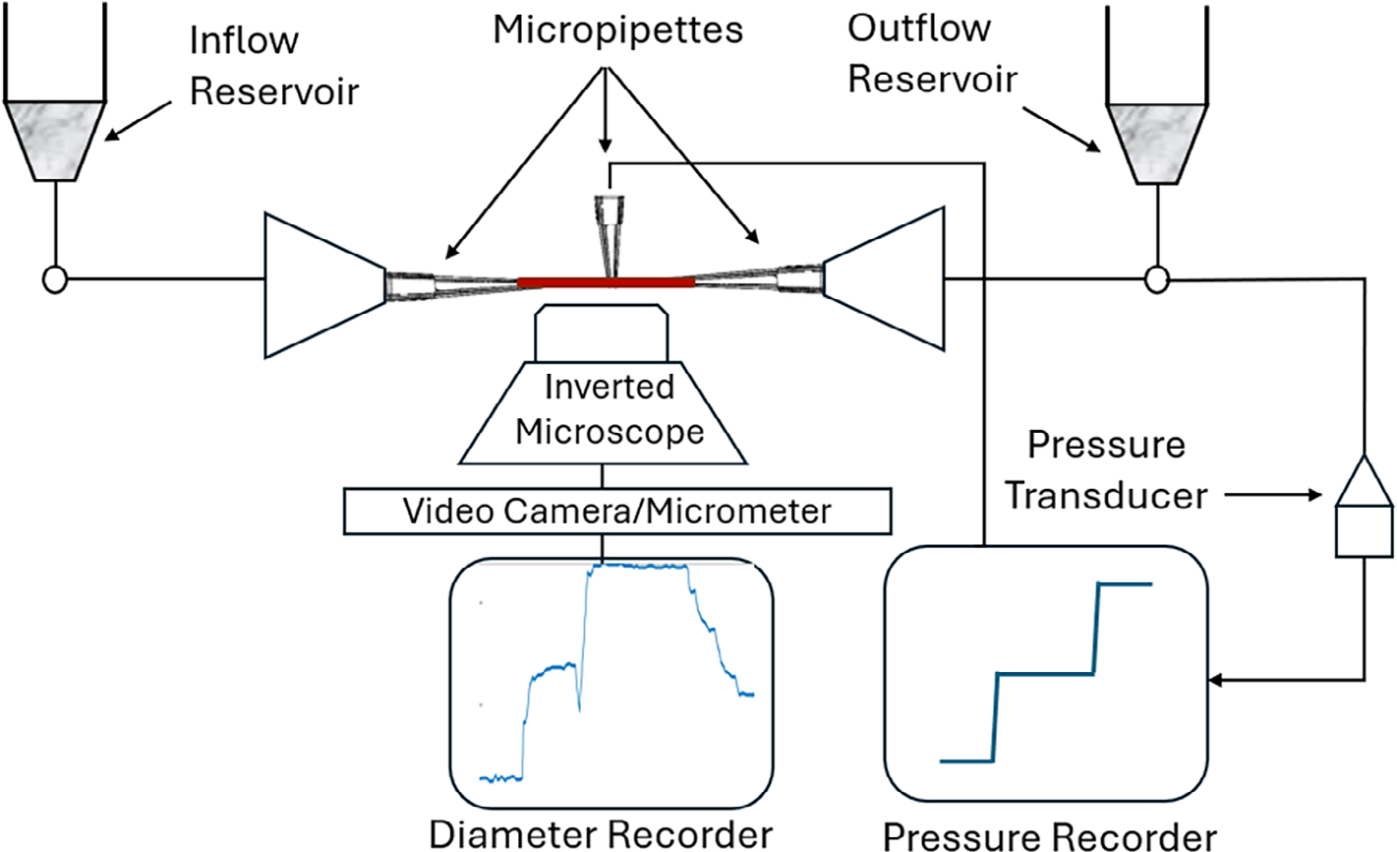
Schematic of the experimental setup by Kuo et al. [12, 13] A dual reservoir system connected to both ends of the cannulated arteriole (which is shown in red) controls the pressure gradient (ΔP) and the intraluminal pressure ($P_{I}$). Upon a change in ΔP, $P_{I}$, or both, recordings of the change in vessel calibre are obtained via microscopic measurements of the internal microvessel diameter. The pressure at the vessel midpoint is also measured to ensure it is equal to the average of the pressure at the two ends. For details see the text.

The experiments by Kuo et al. [12, 13] are, to the best of our knowledge, the only experiments that trace the change in the diameters of the individual arteriole vessels when activating the myogenic and endothelial mechanisms, both in isolation as well as when interacting, in response to changes in the transmural pressure and the pressure gradient. A schematic of the experimental setup is presented in Figure 1. In these experiments:

1. The arteriole vessels are first identified and isolated, and the ends of each arteriole vessel are cannulated and secured with glass micro‐pipettes.
2. Each of the two tips is connected to an independent reservoir system.
3. To initiate the myogenic response: the cannulated arteriole can be pressurized without flow by setting the reservoirs at the two ends to the same hydrostatic level, which can be achieved by simultaneously moving both reservoirs in the same direction, thereby initiating the myogenic response without the endothelial response.
4. To initiate the endothelial response: flow can be initiated by simultaneously moving the reservoirs in opposite directions by the same magnitude thereby generating a pressure difference (ΔP) and initiating the endothelial response without altering the midpoint intraluminal pressure ($P_{I}$).
5. The two mechanisms are examined both under isolation and when interacting with one another by changing the $P_{I}$, ΔP, or both.
6. The corresponding changes in the internal diameter of the cannulated vessel are measured continuously throughout the experiment using video‐microscopic techniques.

A micropipette is also inserted at the midpoint of the cannulated arteriole to ensure that the midline intraluminal pressure is the average of the pressure at the two ends. It should be noted that these experimental data are for porcine coronary autoregulating arterioles; while the use of animal models to try and understand the behavior of human physiology is not without flaws [14], it does provide a useful starting point from which this can be attempted, especially since obtaining in vivo human data is naturally very difficult.

### 1.3 Approximate Bayesian Computation (ABC)

We utilize ABC techniques to calibrate the compliance feedback model parameters. ABC constitutes a class of computational methods rooted in Bayesian statistics that can be used to estimate the posterior distributions of model parameters. Let p be a parameter vector to be estimated. Given some prior distribution $\pi \left(p\right)$, the goal is to approximate the posterior distribution that integrates knowledge from the available experimental data: $\pi \left(p|x\right)\propto f\left(x|p\right)\pi \left(p\right)$, where $f\left(x|p\right)$ is the likelihood of obtaining the data x given the parameter space p [15]. ABC methods have been conceived to infer posterior distributions without having to calculate the likelihood functions, which in many cases can be computationally intractable or too costly to evaluate [15]. Instead, they exploit the computational efficiency of modern simulation techniques by replacing the calculation of the likelihood with a comparison between the measured and simulated data, via some form of MC simulations, which in our case would correspond to the output of the dynamic compliance feedback model. The final parameter space distribution is accordingly informed by the prior distributions and knowledge of the parameters, the model dynamics, and the available experimental data.

The simplest ABC algorithm would be the ABC rejection sampler [16], the general steps for which are summarized below:

1. Sample candidate parameter vector $p^{*}$ from some proposal (prior) distribution $\pi \left(p\right)$.
2. Simulate a dataset $x^{*}$ from the dynamic model, described by a conditional probability distribution $f\left(x|p^{*}\right)$.
3. Compare the simulated dataset, $x^{*}$, with the experimental data, $x_{0}$, using a distance function, d and tolerance ϵ. If $d\left(x_{0},x^{*}\right)\leq \epsilon$, accept $p^{*}$, otherwise reject.
4. Return to step (1) and repeat.

The end result of the simple ABC rejection sampler method is a set of independent samples $p_{1},p_{2},\ldots ,p_{N}$ that approximates the posterior distribution $f\left(p|x\right)$.

The ABC rejection sampler is very easy to implement and relies on the generation of independent realizations and thus can be very easily parallelized. However, the acceptance rate is low when the prior distribution is very different from the posterior distribution [15, 17, 18]. In our model, while some form of a prior distribution can be constructed based on physiological grounds for some parameters like the time constants, the prior distribution for other parameters cannot be approximated a priori. This is especially true when working with multidimensional parameter spaces, in which severe inefficiencies can be generated due to the mismatch between the initial samplings and the target distributions that should meet the desired criteria ($d\left(x_{0},x^{*}\right)\leq \epsilon$). For instance, consider a scenario where a parameter is assigned a high likelihood value; despite this, it may not effectively propagate into the posterior distribution ($d\left(x_{0},x^{*}\right)\geq \epsilon$) if the sampling coincides with a low‐likelihood value for another parameter.

Sampling from the prior probability models is unlikely to be effective when using ABC rejection sampler algorithms in the case where the posterior is a long way from the prior [17]. When such dissimilarity exists, one option that can yield higher acceptance rates is the Markov Chain Monte Carlo (MCMC) scheme, such as the Metropolis‐Hasting algorithm, which work by constructing a Markov Chain whose stationary distribution is the target distribution $\pi \left(p|x\right)$. For likelihood‐free simulations, Marjoram et al. [17] introduced MCMC scheme for generating observations from a posterior distribution without the need for the likelihood calculation, which we call ABC MCMC here. The ABC MCMC scheme (for dynamic systems) is based on the following steps [17].

1. Initialize $p_{i},i=1$.
2. Generate a candidate parameter realization $p^{*}∼q\left(p|p_{i}\right)$ where q is the proposal density, or transition kernel. The simplest transition kernels are normally Gaussian ones with a mean of $p_{i}$ and some prespecified variance.
3. Generate the dataset $x^{*}$ from the dynamic model, which is described by $f\left(x|p^{*}\right)$.
4. Compare the simulated dataset, $x^{*}$, with the experimental data, $x_{0}$, using a distance function, d and tolerance ϵ.
5. If $d\left(x_{0},x^{*}\right)>\epsilon$, go back to step 2, otherwise set and accept $p_{i+1}=p^{*}$ with probability:

(3) $$\alpha =\min\left(1,\frac{\pi \left(p^{*}\right)q\left(p_{i}|p^{*}\right)}{\pi \left(p_{i}\right)q\left(p^{*}|p_{i}\right)}\right)$$

otherwise set $p_{i+1}=p_{i}$.

6 If i<N where *N* is the prescribed Markov Chain length, increment i=i+1 and go to step 2.

Thus, ABC‐MCMC algorithms generate a sequence of serially and highly correlated samples [17] which meet the criteria $d\left(x_{0},x^{*}\right)\leq \epsilon$ after convergence is achieved. The major difference between the conventional and the ABC MCMC methods is that the former requires an explicit definition of the ratio of the likelihood functions to calculate the transition probability upon transition from p to $p^{*}$:

(4) $$\alpha =\min\left(1,\frac{\pi \left(x|p^{*}\right)}{\pi \left(x|p^{*}\right)}\;\frac{\pi \left(p^{*}\right)q\left(p_{i}|p^{*}\right)}{\pi \left(p_{i}\right)q\left(p^{*}|p_{i}\right)}\right)$$

while in the latter case, if both p and $p^{*}$ result in successful realizations ($d\left(x_{0},x^{*}\right)\leq \epsilon$), and, assuming that the perturbation in the parameter values is small, the likelihood ratio is approximated as 1. When the prior distribution is considerably different than the posterior distribution, the ABC‐MCMC algorithm can deliver substantial increases in acceptance rate over ABC rejection sampler algorithm; see [17] for case studies. One disadvantage of such MCMC algorithms, however, is the highly correlated, or dependent, nature of the samples, which, when coupled with low acceptance probabilities for the proposed parameter vector $p_{i}$ or a poor proposal mechanism, can result in long MCMC chains that may get stuck in regions of low probabilities [15, 18]; indeed this is what occurred in this study when attempting to implement an ABC‐MCMC algorithm to calibrate the model parameters characterizing the compliance feedback model.

In theory, the generation of a large number of uncorrelated and independent realizations (or particles) can circumvent this problem of getting stuck in areas of low probability. Furthermore, by generating multiple, uncorrelated realizations starting with a wider prior distribution and a larger error tolerance which then gets gradually narrowed, an ABC algorithm can more efficiently explore higher dimensional parameter spaces without getting stuck in regions of local extrema [15]. This is where sequential Monte Carlo (SMC) ABC schemes come in. First developed by Sisson et al. [18], SMC‐ABC based approaches improve upon the simple rejection sampling method by generating a sequence of independent realizations, or particles, and evolving those through a sequence of intermediary distributions that converge gradually from the prior distribution towards the target distribution. As one moves from one distribution (e.g., at iteration t−1) to the next (at iteration t), the corresponding error tolerance(s) decrease(s). By specifying $\epsilon_{t}<\epsilon_{t-1}$, one ensures that the likely range of parameter values is a subset of the one that precedes it, which is a desirable property for our sampling distribution [15, 18]. A general, simplified version of the SMC‐ABC algorithm is as follows:

1. Set the distribution indicator *t* = 1.
2. Set the error tolerance $\epsilon =\epsilon_{t}$, t=1,…,T such that $\epsilon_{t}<\epsilon_{t-1}$. The error tolerance can be prescribed a priori or heuristically allocated after each distribution iteration.
3. Sample *N* independent parameter realizations/particles $p^{*}$ from the previous distribution $\pi_{t-1}\left(p\right)$ (where $\pi_{0}\left(p\right)$ is the prior distribution).
4. For each particle with parameter realization $p_{i}^{*}$, simulate the candidate dataset $x^{*}$ from the dynamic model, which is described by $f\left(x|p_{i}^{*}\right)$.
5. Compare the simulated datasets, $x_{i}^{*}$, with the experimental data, $x_{0}$, using a distance function, d and tolerance $\epsilon_{t}$: if $d\left(x_{i}^{*},x_{0}\right)\leq \epsilon_{t}$, the corresponding realization $p_{i}^{*}$ is accepted.
6. The accepted realizations are used to create the posterior distribution $\pi_{t}\left(p\right)$.
7. If t<T, set $\pi_{t-1}\left(p\right)=\pi_{t}\left(p\right)$ and t=t+1. Go to step 2.

A more detailed explanation of how the SMC ABC algorithm can be applied in the context of the dynamic compliance feedback model is provided in Section 2.4.1. Note that the algorithm developed by Sisson et al. [18], and later adapted by Toni et al. [15], uses a variation that employs transition kernels and particle weights to resample parameter realizations $\pi_{t+1}\left(p\right)$ from $\pi_{t}\left(p\right)$, rather than explicitly defining a distribution from the accepted realizations and resampling from that new distribution. However, these two approaches are generally equivalent, as they achieve the same outcome. The key features of the SMC ABC scheme, which are the gradual convergence of iterative distributions between the prior and target distributions as the error tolerance is progressively reduced—remain consistent across both variations. Case studies by Sisson et al. [18] and Toni et al. [15] have demonstrated how the SMC approach can significantly outperform both MCMC and rejection sampler ABC methods in terms of accuracy and computational efficiency in particular case scenarios.

Of course, other non‐Bayesian frameworks for parameter calibration exist; one example is the nonlinear mixed effects modeling method (NONMEM) which is widely used in pharmacokinetics, pharmacodynamics, and related fields. NONMEM can be a powerful and effective approach for the parameter calibration of models that involve systems of ODEs and is particularly designed to handle hierarchical data and parameters [19]; for example, those that are shared across the population (known as fixed effects) as well as the variability among individuals that can be modeled with random deviations (known as random effects). In that sense, NONMEM can be suitable in our context because it can capture the fixed or “population effects” that are common across the extracted vessels while also accounting for individual variations between vessels (experiments).

One major difference between approaches like NONMEM which employ a frequentist inference on one hand, and the previously discussed Bayesian approaches, is that the former relies on defining an explicit parameter likelihood function followed by finding the maximum likelihood estimator, which entails the prior designation of parametric distributions, and, particularly in the case of NONMEM, necessitate particular assumptions about the distribution of the random effects and the error structure (the most common of which that they come from Gaussian distributions) [19]. Meanwhile, ABC schemes eliminate the need for a direct likelihood evaluation by using repeated simulated data and distance metrics or statistics to approximate the posterior distribution [18]. When the likelihood function can be explicitly defined and computed, and/or when dealing with standard distributions, frequentist approaches like NONMEM can offer significant advantages in terms of computational efficiency and scalability; however, when the likelihood function is intractable or difficult to compute, the parameter distributions not necessarily well defined, or where assumptions about random effects and error variability cannot be validated, ABC methods offer greater flexibility and may be better suited for those scenarios.

Furthermore, with NONMEM, the numerical solver is embedded with the likelihood estimation, thus requiring gradient‐based methods, which can impose challenges with high‐dimensional and/or multimodal parameter distributions because they can get stuck in regions of local extrema. ABC methods, on the other hand, decouple the numerical solver from the calibration process by first simulating the data from the model and then comparing it to the observed data; for instance, by the use of distance metrics as explained in Section 1.3, and can use multiple independently generated parameter realizations that span the parameter space at each iteration, particularly in SMC ABC schemes. In addition, when compared to ABC methods, NONMEM methods generally lack flexibility when it comes to designating or customizing prior distributions. This could be seen as a disadvantage depending on how accessible and useful prior knowledge of parameter values is. Furthermore, while NONMEM can provide confidence intervals for parameter estimates and thus quantify uncertainty, ABC methods may excel in uncertainty quantification by producing posterior distributions that offer robust assessments of parameter variability, uncertainty quantification, and improved predictive utility.

Ultimately, the choice between a Bayesian framework (like SMC ABC) and a frequentist approach (like NONMEM) depends on several factors: the underlying structure of the model and parameter distributions, the type of available data, the desired level of uncertainty quantification, and the specific context in which these methods are applied. NONMEM generally excels with hierarchical data that incorporates both fixed and random effects, particularly in fields like pharmacokinetics and pharmacodynamics where computational efficiency is crucial, provided that the likelihood is readily computable and relevant assumptions are met. Conversely, Bayesian frameworks are often more suitable for scenarios involving complex or intractable likelihood functions, non‐standard prior distributions or error structures, high‐dimensional or multimodal parameter spaces, or when quantifying uncertainty is vital.

To this end, we first give a brief overview of the experimental data by Kuo et al. used to calibrate the compliance feedback model, along with any necessary preprocessing of the data. We then describe the compliance feedback model adopted from the previous studies [2, 3, 4] as well as the modifications applied to it in this study. Next, we carry out a structural identifiability analysis prior to any data fitting to ensure that the dynamic structure of the model permits the unique identification of the unknown parameter values from the experimental data. We then describe the two‐stage ABC iterative schemes devised and utilized in this study and present the results for each stage, which correspond to the parameter posterior distributions, as well as the comparison between the simulated and the measured diameter traces. Finally, we discuss the results and insights from the parameter calibration approach adopted in this study and offer concluding remarks.

## 2 Methodology

**FIGURE 2:**
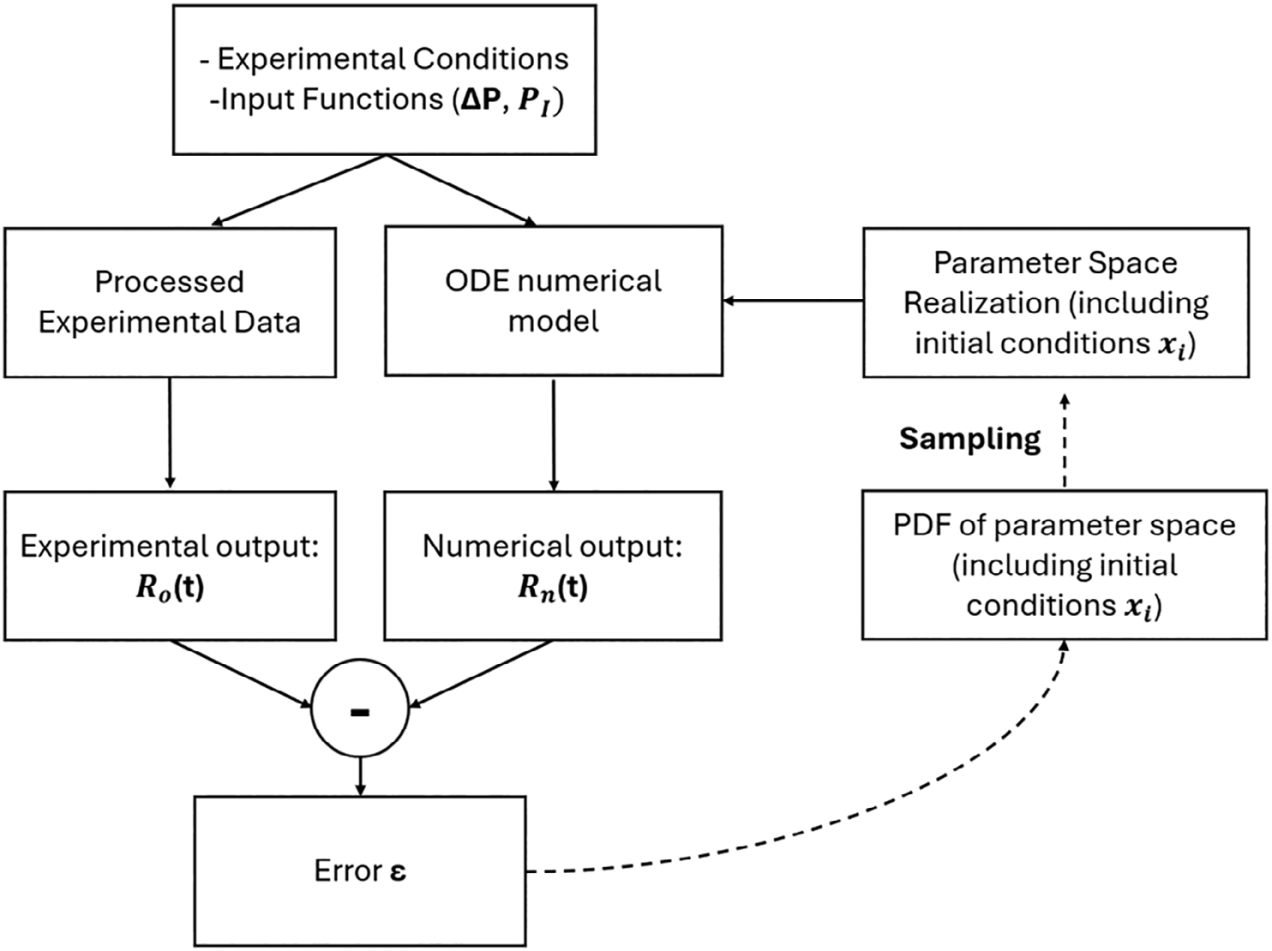
Diagram outlining the approach for the parameter calibration process. The measured (observed) radial traces $R_{o}\left(t\right)$ are obtained from conducting an experiment on a vessel of particular properties and using pre‐specified input conditions ($\Delta P\left(t\right)$ and $P_{I}\left(t\right)$). An in silico simulation utilizing the nonlinear ODE system capturing the vessel dynamics is conducted using the same experimental conditions, along with a particular parameter realization selected from the probability density function (PDF) of the parameter space, to numerically obtain the radial traces $R_{n}\left(t\right)$. The experimentally and numerically derived radial traces are compared, and the difference is used to calculate the error norm, which is in turn used to update the PDF of the parameter space. The process is repeated until PDF convergence or until the maximum number of iterations is reached.

Figure 2 illustrates the methodology employed in this paper for integrating experimental data with the dynamics of numerical models within a framework designed to calibrate the parameters of the dynamic model. The subsequent sections will outline each step and component of this framework in detail.

### 2.1 Preprocessing of Experimental Data

**FIGURE 3:**
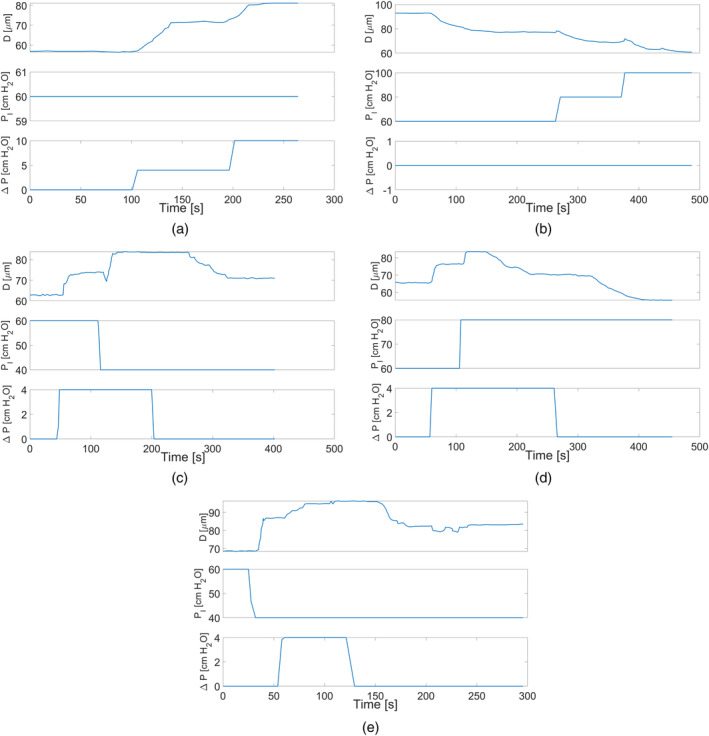
The experimental data, reproduced from the traces by Kuo et al. [12, 13] which includes continuous recordings of the internal diameters (D) of five arteriolar vessels when changing the intraluminal pressure ($P_{I}$) and the pressure difference (ΔP). The data has been deliberately truncated so as not to include any time series points in which the perturbations in $P_{I}$ and ΔP from baseline are greater than 40 cm $H_{2}O$ (30 mmHg) and 10 cm $H_{2}O$ (7.3 mmHg), respectively. The subfigures (a–e) correspond to experiments (1–5).

We reviewed the two studies by Kuo et al. [12, 13] and combined data from a total of six experiments. These experiments involved subjecting six different arteriolar vessels to various combinations of intraluminal pressure and pressure gradient perturbations, and then tracing the changes in the vessel diameter. The isolated arterioles from the two studies have in vivo diameters in the range of 40−80μm, and lengths in the range of 0.7−1.1mm [12, 13]. Since the compliance model is not suitable for large perturbations in pressure and compliance, the experimental data by kuo et al. [12, 13] have been effectively truncated so as not to include any time series points in which the perturbations in $P_{I}$ and ΔP from the baseline value are greater than 40 cm $H_{2}O$ (30 mmHg) and 10 cm $H_{2}O$ (7.3 mmHg), respectively. Also, any step changes in $P_{I}$ and ΔP input functions were slightly smoothed out to circumvent any singularities in the numerical simulation output. Out of the six experimental measurements, five were retained for the purpose of parameter calibration, and one was discarded due to the lack of temporal correlation between the inputs and the output. Figure 3 demonstrates the continuous measurements in internal diameters of the five isolated vessels from the two studies in response to changes in $P_{I}$ and ΔP.

### 2.2 Model Mathematical Formulation

The compliance tube law has been previously used [20, 21, 22, 23, 24, 25, 26] to model the passive alterations (i.e., without autoregulation) in the vessel radius with changes in the transluminal pressure as a result of the vessel compliance. The compliance tube law was first adapted to accommodate the active case where the vessel stiffness can change due to the actions of the dynamic autoregulation mechanisms by Ursino et al. [5] in their compartmental model, where it has successfully reproduced the qualitative effects of the regulatory mechanisms [5]. The compliance feedback model was later utilized in the network models of Daher and Payne [2, 3, 4] to simulate the resulting changes in the calibre of the individual autoregulating vessels. In their model, and for small physiological perturbations from the vessel's baseline conditions [2], the change in the vessel radius can be modelled as:

(5) $$2\mathrm{\pi R}\frac{\mathrm{dR}}{\mathrm{dt}}={\mathrm{CP}}_{I}+{\bar{P}}_{v}\frac{\mathrm{dC}}{\mathrm{dt}}$$

where C is the compliance per unit length, and $C_{b}$ is its baseline value, which can be approximated using the independent ring model [27]. Based on Equation (5), we distinguish between two vessel types. Nonregulating vessels have a constant compliance, and hence only react passively to changes in $P_{I}$. On the other hand, in regulating vessels, C is dynamically modified by the actions of the autoregulatory control mechanisms. Hence, the change in the vessel cross‐sectional area can be ascribed to both the passive response to $P_{I}$ changes and the action of control mechanisms on C. The compliance of the vessel can be altered within limits due to the myriad autoregulatory mechanisms, which, in our study, include the myogenic and endothelial mechanisms. The effects of the myogenic and endothelial responses can be modelled via activation factors, $x_{m}$ and $x_{e}$, respectively, which can then be nonlinearly mapped into the vessel compliance, as proposed previously [2, 3, 4, 5] via a non‐symmetrical sigmoidal function to capture the autoregulatory effects on the vessel compliance:

(6) $$C=C_{b}\left[1+\frac{\Delta C^{\pm }}{C_{b}}\tan h\left(\frac{1}{G}\frac{C_{b}}{\Delta C^{\pm }}\left(x_{e}-x_{m}\right)\right)\right]$$

where $\Delta C^{\pm }$ are the maximal and minimal fractional changes in compliance, and the parameter G dictates the central slope between C and $\left(x_{e}-x_{m}\right)$. The myogenic and endothelial activation factors ($x_{m}$ and $x_{e}$) are altered in response to perturbations in the vessel wall tension (which itself depends on the intraluminal pressure), as well as the shear stress. Hence, a linear feedback model emulating first‐order low‐pass filter dynamics can be assumed for each of the myogenic ($x_{m}$) and endothelial ($x_{e}$) activation factors [4]:

(7) $$\frac{{\mathrm{dx}}_{m}}{\mathrm{dt}}=\frac{1}{\tau_{m}}\left(-x_{m}+S_{m}\left(\text{ReLU}\left(\frac{{\mathrm{RP}}_{I}-T_{0}}{T_{0}}\right)\right)\right)$$

(8) $$\frac{{\mathrm{dx}}_{e}}{\mathrm{dt}}=\frac{1}{\tau_{e}}\left(-x_{e}+S_{e}\left(\text{ReLU}\left(\frac{R\left(t-d\right)\Delta P\left(t-d\right)-\tau_{0}}{\tau_{0}}\right)\right)\right)$$

where $\tau_{m}$ and $\tau_{e}$ are the time constants of the myogenic and endothelial mechanisms, respectively. S is the sensitivity, or gain factor, for the perturbations of each of the wall tension ($T={\mathrm{RP}}_{I}/2$) or shear stress (τ=RΔP/2L) from their reference values. In this model, the activation term for each of the two responses depends on the perturbation from a reference value of tension $T_{0}$ and shear value $\tau_{0}$ (equal to the shear stress divided by 2L), respectively, at which the myogenic or endothelial gain factor would be zero. Furthermore, to ensure that no activation (or deactivation) happens below such reference values, the perturbation signal for each of the two responses first goes through the ReLU function before being amplified by the gain factor. Finally, as seen in Figure 3b–e, we notice that when endothelial‐induced constriction takes place as a result of a drop in ΔP, there is a delay before the constriction takes place (for Figure 3b, the endothelial constriction is postulated to occur as a result of removing the vessel from an environment where shear stress was present, to the experimental setup where ΔP=0). This delay can be hypothesized (though in no way confirmed) to occur as a result of the presence of lingering nitric oxide endothelial factor that takes time to degrade [28] or wash away through advection and diffusion when the shear stress acting on the endothelial layer decreases. Regardless of the underlying physiological basis, this delay in activation (d) can be simply mathematically described as:

(9) $$d=\left\{\begin{array}{cc} \zeta & \frac{\mathrm{d\tau}}{\mathrm{dt}}<0\\ 0 & \text{otherwise} \end{array}\right.$$

where ζ is the delay in seconds preceding the endothelial‐induced constriction (i.e., when shear stress decreases). We differentiate Equation (6) with respect to time to obtain an expression for $\frac{\mathrm{dC}}{\mathrm{dt}}$ in terms of $x_{e}$ and $x_{m}$, as well as their rates of change, $\frac{{\mathrm{dx}}_{m}}{\mathrm{dt}}$ and $\frac{{\mathrm{dx}}_{e}}{\mathrm{dt}}$, the expressions for which can be derived from Equations (7) and (8). The expression for $\frac{\mathrm{dC}}{\mathrm{dt}}$ can then be inserted into Equation (5), to obtain the first‐order differential equation for the rate of change of the internal radius:

(10) $$\begin{array}{c} \frac{\mathrm{dR}}{\mathrm{dt}}=\frac{1}{2\mathrm{\pi R}}\left[C_{b},\left(1+\frac{\Delta C^{\pm }}{C_{b}}\tan h\left(\frac{C_{b}}{G\Delta C^{\pm }}\left(x_{e}-x_{m}\right)\right)\right),\frac{{\mathrm{dP}}_{I}}{\mathrm{dt}}\right.+{\bar{P}}_{v}\frac{C_{b}}{G}\sec h^{2}\left(\frac{C_{b}}{G}\left(x_{e}-x_{m}\right)\right)\\ \left.\cdot \left(\frac{x_{m}}{\tau_{m}}-\frac{S_{m}}{\tau_{m}}\left(\text{ReLU}\left(\frac{{\mathrm{RP}}_{I}-T_{0}}{T_{0}}\right)\right)-\frac{x_{e}}{\tau_{e}}+\frac{S_{e}}{\tau_{e}}\left(\text{ReLU}\left(\frac{R\left(t-d\right)\Delta P\left(t-d\right)-\tau_{0}}{\tau_{0}}\right)\right)\right)\right] \end{array}$$

Table 1 provides a summary of the variables involved in the nonlinear Equations (7), (8), and (10), which constitute our dynamic system. The table includes the variable type (state variable, input variable, fixed parameter, or unknown parameter) along with a brief description of each variable. For more information about the starting or nominal values of these variables and their sources, see Section 2.4.1.

**TABLE 1:** The variables in the system of nonlinear differential Equations in (7), (8), and (10), along with their types, (SI) units, and a brief description of each. Free parameters are the ones whose values are to be calibrated, while fixed parameters are treated as constant (see Section 2.4.1). Input variables are those which are changed throughout the course of the experiment to initiate the myogenic and endothelial responses.

Variable	Type	Brief description	Unit
$x_{m}$	State variable	Myogenic activation factor	—
$x_{e}$	State variable	Endothelial activation factor	—
R	State variable	Vessel internal radius	m
$\tau_{m}$	Free parameter	Myogenic time constant	s
$\tau_{e}$	Free parameter	Endothelial time constant	s
$S_{m}$	Free parameter	Myogenic sensitivity/gain factor	—
$S_{e}$	Free parameter	Endothelial sensitivity/gain factor	—
$T_{0}$	Free parameter	Reference vessel wall tension at which no myogenic activation occurs	$\mathrm{kg}/s^{2}$
$\tau_{0}$	Free parameter	Reference shear stress (normalized by vessel length) at which no endothelial activation occurs	$\mathrm{kg}/s^{2}$
G	Free parameter	Sets the central slope of C as a function of ($x_{e}-x_{m}$)	—
d	Free parameter	Delay in endothelial‐induced constriction	s
$C_{b}$	Fixed parameter	Baseline vessel compliance (per unit length)	$m^{3}s^{2}/\mathrm{Kg}$
$\frac{\Delta C^{\pm }}{C_{b}}$	Fixed parameter	Maximal and minimal fractional changes in compliance	—
$P_{I}$	Input variable	Intraluminal pressure of the vessel	Pa
ΔP	Input variable	Pressure gradient across the vessel	Pa
t	Independent variable	Time	s

Equations (7), (8), and (10) form a system of three first‐order nonlinear ODEs in terms of the system variables x:

(11) $$\frac{dx}{\mathrm{dt}}=f\left(x,p,u\right)$$

where $x={\left[R,x_{m},x_{e}\right]}^{T}$ are the system variables, $p={\left[S_{m},S_{e},G,T_{0},\tau_{0},\tau_{m},\tau_{e},d\right]}^{T}$ are the eight unknown model parameters, and $u={\left[I_{P},\Delta P\right]}^{T}$ is the input vector for the experimental setup. Note that from the state variables x, only R is measured. Furthermore, to account for errors in the radial measurements and uncertainties in the state variables, the initial conditions $x_{i}={\left[R_{i},x_{\mathrm{mi}},x_{\mathrm{ei}}\right]}^{T}$ are also assumed to be unknown and are concatenated with the parameter vector p to obtain the vector of variables V to be calibrated.

The nonlinear system of equations is solved in MATLAB using a first‐order hybrid implicit‐explicit (IMEX) Euler scheme with a timestep of Δt=0.2s. To obtain a discrete representation of the diameter traces, data points are sampled from the continuous measurement tracings at 1.5‐s intervals. The experimental input functions (ΔP and $P_{I}$), defined as step changes, are reconstructed by identifying the times at which these changes occur. These input functions are then linearly interpolated (using MATLAB's interp1 function) to match the time points of the numerical scheme and are subsequently smoothed. The derivative dP/dt is calculated from the interpolated $P_{I}$ using MATLAB's setdiff function. Finally, the simulated diameter values generated by the numerical scheme are sampled at 1.5‐s intervals to enable direct comparison with the experimental data.

### 2.3 Structural Identifiability Analysis

Numerous techniques have been developed to interrogate and examine the structural identifiability of linear and nonlinear systems of equations; see [10] for a discussion of the advantages and disadvantages of each method. Since our model can be formulated as a system of nonlinear ODEs, with a linear dependency on the input functions (see Equation 14) the generating series approach is used, which offers the best compromise between applicability, computational complexity, and information provided [10]. If the solution of the system of equations is unique, the parameters are globally identifiable. We use the Generating Series for Testing Structural Identifiability (GenSSI) MATLAB toolbox developed by Chis et al. [9], which utilizes automatic symbolic computations and combines the use of the generating series approach with the use of identifiability tableaus [29] to assess the structural identifiability of any arbitrary nonlinear model of biological systems. To use the GenSSI toolbox, each linear/nonlinear differential equation in the system must be written in the form below:

(12) $$\frac{\mathrm{dx}\left(t\right)}{\mathrm{dt}}=f\left(x\left(t\right),p\right)+\sum_{i=1}^{m}g\left(x\left(t\right),p\right)u_{i}\left(t\right)\;x\left(t_{0}\right)=x_{0}\left(p\right)$$

and the observable(s) in the form:

(13) $$y\left(t,p\right)=h\left(x\left(t\right),p\right)$$

where $x\in R^{n}$ is the n‐dimensional state variable, $u\in R^{m}$ is an n‐dimensional control, $y\in R^{r}$ is the r‐dimensional output, which, in our case, is the internal diameter of the vessel, and $x_{0}\left(p\right)$ are the initial conditions. The model response will depend on a number of parameters $p\in P\subseteq R^{q}$, some of which can be fixed while the others would be unknown.

The underlying idea behind the generating series approach is to generate a nonlinear system of equations in terms of the parameters with unknown values from the successive Lie derivatives of the observable vector field $h\left(x\left(t\right),p\right)$ along the nonlinear vector fields (f and g in Equation (14)), and to determine if there is a unique solution to the system of equations in terms of the parameters. In addition, the use of identifiability tableaus, which represent the non‐zero elements of the Jacobian of the series coefficients with respect to the parameters can help visualize the possible structural identifiability problems [10], as outlined in Section 3.1.

We note that in all the simulations conducted in this study (and in general for perturbations around the baseline conditions), the argument of the RELU function in Equations (7), (8), and (10) is positive because the shear stress and vessel wall tension do not reach the reference point at which the myogenic and endothelial responses are no longer activated. In other words, the omittance of the RELU function does not affect the outputs in this study. Hence, to avoid mathematical intricacy and any discontinuities in the derivatives, we omit the dependence on the RELU function for the sake of the structural identifiability analysis. Secondly, and again for mathematical simplicity, we omit the delay in the endothelial‐induced constriction in Equation (8) when conducting the structural identifiability test to ensure that the Equations are of the form in Equation (12). Hence, we end up two parameters (${\left[C_{b},\Delta C^{\pm }\right]}^{T}$) that are fixed, and seven parameters (${\left[\tau_{m},\tau_{e},S_{m},S_{e},T_{0},\tau_{0},G\right]}^{T}$) to test. The vector of the state variables in Equation (12) is the same as before, and the output function (the observable quantity) can be defined in terms of the state variables as y=2R. We choose our input functions (those which are altered during the experiment) to be ${\left[\Delta P,P_{I},\frac{{\mathrm{dP}}_{I}}{\mathrm{dt}}\right]}^{T}$, where, because of the linear dependency of the input functions in Equation (12) required to conduct the analysis on GenSSI, we have added $\left(\frac{{\mathrm{dP}}_{I}}{\mathrm{dt}}\right)$ as an extra input function (even though it can be directly deduced from $P_{I}$). Hence, our dynamic model can now be written in the form:

(14) $$\frac{dx}{\mathrm{dt}}=f\left(x,p\right)+u^{T}G\left(x,p\right)$$

where f is a vector of length n=3 and is given by:

(15) $$f=\left[\begin{array}{c} -\frac{1}{\tau_{e}}\left(x_{e}+S_{e}\right)\\ -\frac{1}{\tau_{m}}\left(x_{m}+S_{m}\right)\\ \frac{\bar{P_{v}}C_{b}}{2\mathrm{\pi RG}}\sec h^{2}\left(\frac{C_{b}G}{\Delta C^{\pm }}\left(x_{e}-x_{m}\right)\right)\left(\frac{1}{\tau_{m}}\left(x_{m}+S_{m}\right)-\frac{1}{\tau_{e}}\left(X_{e}+S_{e}\right)\right) \end{array}\right]$$

and G is an n×m=3×3 matrix corresponding to the coefficients of the input functions:

(16) $$G=\left[\begin{array}{ccc} a_{11} & 0 & 0\\ 0 & a_{22} & 0\\ a_{31} & a_{32} & a_{33} \end{array}\right]$$

where the entries of the matrix are given by:

(17) $$a_{11}=\frac{S_{e}R}{\tau_{e}\tau_{0}}$$

(18) $$a_{22}=\frac{S_{m}R}{\tau_{m}T_{0}}$$

(19) $$a_{31}=\frac{\bar{P_{v}}C_{b}S_{e}R}{2\mathrm{\pi RG}\tau_{e}{\mathrm{tau}}_{0}}\sec h^{2}\left(\frac{C_{b}}{G\Delta C^{\pm }}\left(x_{e}-x_{m}\right)\right)$$

(20) $$a_{32}=-\frac{\bar{P_{v}}C_{b}S_{m}R}{2\mathrm{\pi RG}\tau_{m}T_{0}}\sec h^{2}\left(\frac{C_{b}}{G\Delta C^{\pm }}\left(x_{e}-x_{m}\right)\right)$$

(21) $$a_{33}=\frac{C_{b}}{2\mathrm{\pi R}}\left(1+\frac{\Delta C^{\pm }}{C_{b}}\tan h\left(\frac{C_{b}}{G\Delta C^{\pm }}\left(x_{e}-x_{m}\right)\right)\right)$$

Once the equations are inserted into the GenSSI toolbox in the format of Equation (12), the toolbox can then assess the structural identifiability of each parameter as globally structurally identifiable, locally structurally identifiable, or structurally non‐identifiable, as previously described in Section 1.1.

### 2.4 Algorithm for the First‐Stage ABC

#### 2.4.1 Algorithm for the First‐Stage ABC

Building on the general methodology for ABC SMC algorithms outlined in Section 1.3, we have designed an iterative SMC algorithm to calibrate the parameters of the model defined by the system of nonlinear ODEs in Equation (11). This algorithm employs sequential narrowing of tolerances and convergence of parameter distributions in line with the SMC features. An outline of the first‐stage algorithm is provided below.

1. Initialize the iterative process:
  - Set the number of iterations, r.
  - Define the initial prior distribution, $\pi_{V}$.
  - Set the initial error tolerances ($\epsilon_{2}$, $\epsilon_{4}$, and $\epsilon_{\infty }$).
2. Iterate over r steps (*i* = 1, …, *r*):
  - Sample MC realizations from the prior distribution to create the vector space V.
3. Evaluate realizations:
  - For each realization j in MC:
    ○ Generate the diameter trace $D_{j}$ by numerically integrating the dynamic model in Equation (11) using experimental inputs $P_{I}\left(t\right)$ and $\Delta P\left(t\right)$ and parameter space realization $V_{j}$.
    ○ Compare the simulated diameter $D_{j}$ trace to the observed data trace $D_{0}$ by calculating the error norms: $L_{2}$ if r=1, and $L_{2},L_{4},\text{and}\;L_{\infty }$ otherwise.
4. Apply acceptance criteria (for each of the MC realizations)
  - For the first iteration (i=1):
    ○ Accept the realization if the two‐error norm $L_{2}\left(D_{j},D_{0}\right)$ is within $\epsilon_{2}$.
  - For subsequent iterations (i>1):
    ○ Accept the realization if all error norms ($L_{2}$, $L_{4}$, and $L_{\infty }$) meet their respective tolerances.
5. Update the posterior distribution
  - Fit a posterior distribution $\pi_{V}^{\mathrm{new}}$ using the accepted realizations.
  - Set the new posterior as the prior for the next iteration ($\pi_{V}=\pi_{V}^{\mathrm{new}}$).
6. Adjust error tolerances
  - Gradually decrease the error tolerances ($\epsilon_{2}$, and $\epsilon_{4}/\epsilon_{\infty }$ for i>1) to refine the accuracy of accepted realizations.

In the algorithm above, r is the number of iterations (set here to 5) and MC is the number of realizations created (set here to 1e5). For i=1, The starting distribution $\pi_{V}$ for the variable space is the prior distribution of the parameter space, which is outlined in Section 2.4.1. For the sake of simplicity, we assume that the experimental inputs $P_{I}\left(t\right)$ and $\Delta P\left(t\right)$ have no associated errors. For the first iteration $\left(i=1\right)$, the $L_{2}$ (Euclidean) norm is calculated between the simulated dataset $D_{j}$ and the measured dataset $D_{0}$, and it must be below the error tolerance $\epsilon_{2}$ for the realization to be accepted. For the remaining iterations, the $L_{4}$ and $L_{\infty }$ norms are also calculated and must also be below certain thresholds to accept the realization, as they are effective in limiting larger discrepancies at individual time points, leading to a more refined set of realizations overall.

#### 2.4.2 Initialization of the Prior Distributions

The ABC algorithm described above requires an initial prior distribution of the parameters during the first iteration (*i* = 1). Table 2 summarizes the initial conditions assigned to the model parameters and the physiological motivations behind such assignments, which are further described in this section. Since we want a higher sampling likelihood around the mean values, the initial distribution of all parameters (except $x_{\mathrm{ei}}$) is assumed to be Gaussian ($N\left(\mu ,\sigma^{2}\right)$), which requires two parameters to be specified, the mean (μ) and the standard deviation (σ). The mean for the initial radii is set to $\mu =D_{0}\left(0\right)/2$ (i.e., the experimentally measured initial radius) and the standard deviation is set (arbitrarily) to σ=5μm. Since the myogenic response has been reported to occur faster than the endothelial response [30, 31, 32], the initial distributions of the time constants are set to $\tau_{m}∼N\left(18\;s,100\;s^{2}\right)$ and $\tau_{e}∼N\left(40\;s,400\;s^{2}\right)$ in line with the range of values from the literature [12, 32, 33, 34]. The distributions for the gain factors of the myogenic and endothelial responses are initialized to $S_{m}∼N\left(4,4\right)$ and $S_{e}∼N\left(1,0.56\right)$, such that the values for the mean are the same as those used in in the previous studies [2, 3, 4]. In these previous models, the parameter values were chosen after analyzing the model output based on an overall understanding of the behavior of the network physiological response, with $S_{m}$ chosen to be greater than $S_{e}$ due to the myogenic dominance reported in other studies [35, 36]. Similarly, the mean value for G was chosen from the previous model simulations, to give a distribution of $G∼N\left(0.1,0.009\right)$.

**TABLE 2:** The initial values and distributions of the model parameters, and the corresponding sources. $N\left(\mu ,\sigma^{2}\right)$ indicates a Gaussian distribution with mean μ and standard deviation σ.

Parameter	Type	Prior distribution	Source
$R_{i}$	Initial condition	$N\left(D_{0}/2.25{\left(\mathrm{\mu m}\right)}^{2}\right)$	The mean is set to the initial radius reading $D_{0}/2$.
$x_{m,i}$	Initial condition	$N\left(x_{m,0},1\right)$	The mean is approximated from iterative numerical simulations until steady‐state conditions equivalent to baseline conditions are reached.
$x_{e,i}$	Initial condition	$N\left(x_{e,0},0.56\right)$. For experiments with $X_{e,0}=0$, half‐normal distributions are used	
$S_{m}$	Free parameter	$N\left(4,4\right)$	Values for the mean are taken from previous numerical studies [2, 3, 4] based on an understanding of the expected physiological behavior.
$S_{e}$	Free parameter	$N\left(1,0.56\right)$	
G	Free parameter	$N\left(0.1,0.009\right)$	
$T_{0}$	Free parameter	Microvessel (experiment) dependent	Approximated as half the baseline wall tension.
$\tau_{0}$	Free parameter	Microvessel (experiment) dependent	Approximated as half the baseline wall shear stress (normalized by 2L).
d	Free parameter	$N\left(33s,100s^{2}\right)$	Calculated by measuring the delay in experiments involving endothelial‐induced constriction.
$\tau_{m}$	Free parameter	$N\left(18s,100s^{2}\right)$	The means are identified from previous studies [12, 32, 33, 34].
$\tau_{e}$	Free parameter	$N\left(40s,400s^{2}\right)$	
$C_{b}$	Fixed Parameter	Vessel (experiment) dependent	Calculated from the radius and estimated vessel properties (thickness, Young's modulus) using the independent ring's model [27]. Fixed due to minimal impact on output according to previous sensitivity analyses [2, 3].
$\Delta C^{+}$	Fixed parameter	10	Taken directly from Ursino's model [34] and used in subsequent studies [2, 3, 4]. Fixed due to minimal impact on output according to previous sensitivity analyses [2, 3].
$\Delta C^{-}$	Fixed parameter	0.8	

The mean values for reference values of tension ($T_{0}$) and normalized shear stress ($\tau_{0}$) at which no activation occurs were chosen to be equal to half of the baseline values of the vessel wall tension and shear stress, respectively, for each vessel. The baseline values for tension and shear stress were extracted as follows: in the experiments by Kuo et al. [12, 13], the baseline value for $P_{I}$ reflecting in situ conditions for vessels of such diameter and length range was reported by the authors of the study to be $60\;\mathrm{cm}\;H_{2}O=44.13\;\text{mmHg}$ (which is also the value for ${\bar{P}}_{v}$ in Equation (10)). The baseline (in situ) value for the ΔP was set to 5mmHg by extrapolating the data by Payne and Lucas [37], which has listed the pressure drop across the arteriolar microvessels based on their lengths and internal diameter. The distribution mean for the constrictive endothelial response delay (ζ) was determined by calculating the delay across the experiments involving endothelial‐induced constriction, which is the time between the point at which ΔP is decreased, and the point when endothelial‐induced constriction starts, which was then averaged across the different experiments involving endothelial‐based constriction, resulting in a distribution of $\zeta ∼N\left(33\;s,100\;s^{2}\right)$.

The initial values for $x_{m}$ and $x_{e}$ (i.e., for *i* = 1) for each experiment were obtained by iteratively solving the ODE system in Equation (11) under the conditions $P_{I}\left(t=0\right)$ and $\Delta P\left(t=0\right)$. The initial values for $x_{m}$ and $x_{e}$ were updated in each iteration until they converged, ensuring that the steady‐state value of $R_{0}\left(0\right)$ was achieved; that is, until the numerical solver both started with and maintained the value of $R_{0}\left(0\right)$. Note that this is only relevant for the initial values, since, upon successive calibration, the initial conditions that give rise to simulations that better fit the observed data would not necessarily correspond to steady‐state initializations. The initial values were used as the mean, with standard deviations of 1 and 0.75 for $x_{m}$ and $x_{e}$, respectively. For all experiments with $\Delta P\left(0\right)=0$, $\mu \left(x_{\mathrm{ei}}\right)$ was set to zero and the distribution was modelled using a half‐normal distribution (i.e., ignoring the negative values). Finally, only for the second experiment in Figure 3b, an additional parameter $\tau_{0,\text{hist}}$, also calculated from the initial steady‐state baseline conditions, was initialized and added to the variable space as a parameter to be calibrated. This parameter corresponds to the value of the normalized shear stress before starting the measurement recording $\left(\tau \left(t<0\right)\right)$, since at t=0, ΔP drops, giving rise to a delayed endothelial‐induced constriction, which, according to Equation (10) requires prior knowledge of $\tau \left(t<0\right)$. The distributions were chosen such that it is highly unlikely for a random variable to have a value <0; nonetheless, a filtering criterion was added to prevent negative value realizations.

We also note that we have treated the maximal and minimal fractional change in compliance parameters $\left(\frac{\Delta C^{\pm }}{C_{b}}\right)$, as well as the baseline compliance, $C_{b}$ as fixed since previous parameter sensitivity analyses of similar models [2, 3] revealed relatively little influence of these parameters on the model outputs (which were the cerebral blood flow and volume in a network of microvessels). The values for $\left(\frac{\Delta C^{\pm }}{C_{b}}\right)$ were set to 10 and 0.8, respectively, based on the values from a previous compliance feedback model [34], and the value of $C_{b}$ was determined from the independent ring model [27].

After the realizations are accepted or rejected, and the space of accepted realizations $V_{k}$ is compiled, MATLAB's DistributionFitter application from the Statistics and Machine Learning toolbox is used to fit a univariate distribution to $V_{k}$. In rare cases, when no univariate distribution was deemed fit, a kernel distribution, defined by a smoothing function and a bandwidth value that controls the smoothness of the fitting curve, was used to fit the data. The parameters characterizing the posterior fitted distribution are then used to generate the variables space V for the next iteration from which the realizations are sampled. The error tolerances are appropriately decreased for the next iteration, the exact value by which is heuristically determined based on the calculated L‐norms of the accepted realizations $V_{k}$. The algorithm is first applied independently for each of the five experiments involving five different vessels, such that for each experiment, final posterior distributions for the parameters and initial conditions are obtained.

### 2.5 Second‐Stage ABC Scheme

The ABC scheme aims to obtain a single final probability distribution of the model parameters. The final distributions obtained from the first stage relate to each experiment (i.e., vessel) and the corresponding input functions individually. Due to the interaction and nonlinear effects between the model parameters, as examined in the previous studies [2, 3, 4], the final distributions for the model parameters cannot be simply combined across the different experiments to obtain a single distribution representative of all experiments. The final distribution for the model parameters must fit all relevant experiments, meaning that a single realization of the parameter space must simultaneously lie within the prescribed error tolerance across all the relevant experiments for it to be accepted. To this end, we carry out a second round of an iterative ABC scheme as described below.

First, we divide the experiments, the measurements for which are demonstrated in Figure 3, into two groups: group A encompassing experiments (1, 3, and 5), where the myogenic response is dilation‐based, and group B encompassing experiments (1, 2, and 4) in which the myogenic response results in constriction. The reason for that is that the first‐stage ABC scheme showed significant differences in the time constants between myogenic‐induced constriction and dilation (see Section 3). Note that both groups include experiment 1, which does not feature any significant myogenic response ($P_{I}$ is kept constant), but is rather used to help calibrate the parameters of the endothelial response along with the other experiments in each group.

Within each group, variables that are specific to the individual experimental setup (the initial conditions ${\left[R_{i},x_{\mathrm{mi}},x_{\mathrm{ei}}\right]}^{T}$) are treated independently and are sampled from the final posterior distribution of the first‐stage ABC in Section 2.4.1. Meanwhile, variables that are shared (all the parameter space p) are sampled from a single distribution. The prior distribution for the shared parameters is initially constructed by sampling from the fitted final posterior distributions from Section 2.4.1 with an equal probability across the three experiments within each group. Note that for the first iteration, the final posterior distributions of the parameters from the first‐stage ABC of the myogenic response ($T_{0},\tau_{m},\text{and}\;S_{m}$) for experiment 1 are not used in constructing the prior distribution in second‐stage ABC, since the myogenic response is not directly induced in that experiment.

Next, we devise an iterative ABC algorithm, where, just as in the first‐stage in Section 2.4.1, a variable space realization is accepted only if the $L_{2}$, $L_{4}$, and $L_{\infty }$ norms across all three experimental measurements and simulation outputs are within the prescribed error tolerances; these, in turn, being gradually narrowed across the iterations. Again, just as for the first‐stage ABC, the fitted posterior distributions for a particular iteration become the prior distribution for the next iteration from which the variable space is sampled. The algorithm for the second‐stage iterative SMC ABC scheme is described below (where, again, the number of iterations (r) is set to 5 and the number of realizations generated during each iteration (MC) is set to 1e5).

1. Initialize parameters
  - Set the number of iterations r.
  - Define the initial prior distribution $\pi_{V}$.
  - Set the error tolerances $\epsilon_{2},\epsilon_{4},\epsilon_{\infty }$.
2. Iterative process
  - For each iteration i in r:
    - Sample initial vector space
      – Generate MC realizations from the prior $\pi_{V}$, resulting in a vector space V.
      – Initialize an empty list of accepted realizations, accepted_V.
    - Evaluate realizations
      – For each realization j in V:
        * Simulate the first dataset $D_{j,1}$ using the dynamic model and experimental inputs $P_{I,1},\Delta P_{1}$ and compare to experimental measurements ($D_{1}$).
        * If all error norms for $D_{j,1}$ are within tolerances ($\epsilon_{2},\epsilon_{4},\epsilon_{\infty }$):
          - Simulate the second dataset $D_{j,2}$ using inputs $P_{I,2},\Delta P_{2}$ and compare to experimental measurements ($D_{2}$).
          - If all error norms for $D_{j,2}$ are within tolerances:
            ○ Simulate the third dataset $D_{j,3}$ using inputs $P_{I,3},\Delta P_{3}$ and compare to experimental measurements ($D_{3}$).
            ○ If all error norms for $D_{j,3}$ are within tolerances:
            ○ Accept realization j and add it to accepted_V.
    - Update posterior distribution
      – Fit a posterior distribution $\pi_{V}^{\mathrm{new}}$ using the accepted realizations accepted_V.
      – Set $\pi_{V}=\pi_{V}^{\mathrm{new}}$ for the next iteration.
      – Decrease error tolerances ($\epsilon_{2},\epsilon_{4},\epsilon_{\infty }$).

## 3 Results

### 3.1 Structural Identifiability Analysis

**FIGURE 4:**
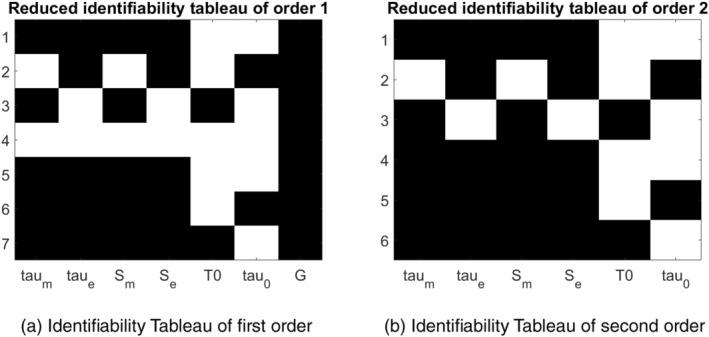
Reduced identifiability tableau of order 1 (a) and 2 (b), illustrating the structural identifiability of model parameters using the first‐order derivatives (a), and when incorporating second‐order derivatives to refine the identifiability analysis (b) of the system equations. The first‐order analysis revealed that the parameter G was globally identifiable, and thus can be eliminated from the analysis in the second‐order tableau.

The model formulation described in Section 2.3 was analyzed using the GENSSI program for structural identifiability assessment. GENSSI first computes the first‐order Lie derivatives of the observable function D=2R along the vector fields f and g in Equation (14). From the first‐order derivative, a symbolic expression can be directly determined only for the parameter G, indicating that G is globally structurally identifiable at this stage. To evaluate the identifiability of the remaining parameters, the rank of the Jacobian matrix (constructed from the first‐order derivatives) is considered. Since the rank is 4 (less than the full rank of 7, which corresponds to the total number of parameters), the first‐order derivatives alone are insufficient to prove the unique identifiability of all the parameters. Consequently, the second‐order Lie derivatives are computed, resulting in a Jacobian rank of 7, which confirms that the remaining parameters are at least locally identifiable. Once local identifiability is established, the GENSSI algorithm applies an iterative procedure to solve the system of equations for the remaining parameters [29]. Through this process, explicit symbolic expressions are derived for all parameters, confirming that the model parameters are globally structurally identifiable. Figure 4 presents the reduced identifiability tableaus for the first and second orders. These tableaus represent the non‐zero elements of the Jacobian matrix with respect to the parameters. Each tableau has columns corresponding to parameters and rows corresponding to the Lie derivatives for a given order: a black square at position i,j indicates that the corresponding ith non‐zero generating series coefficient depends on the parameter in position j. The absence of empty columns indicates that no parameters are unidentifiable, a conclusion supported by the symbolic computations.

### 3.2 Results of the First‐Stage ABC

**FIGURE 5:**
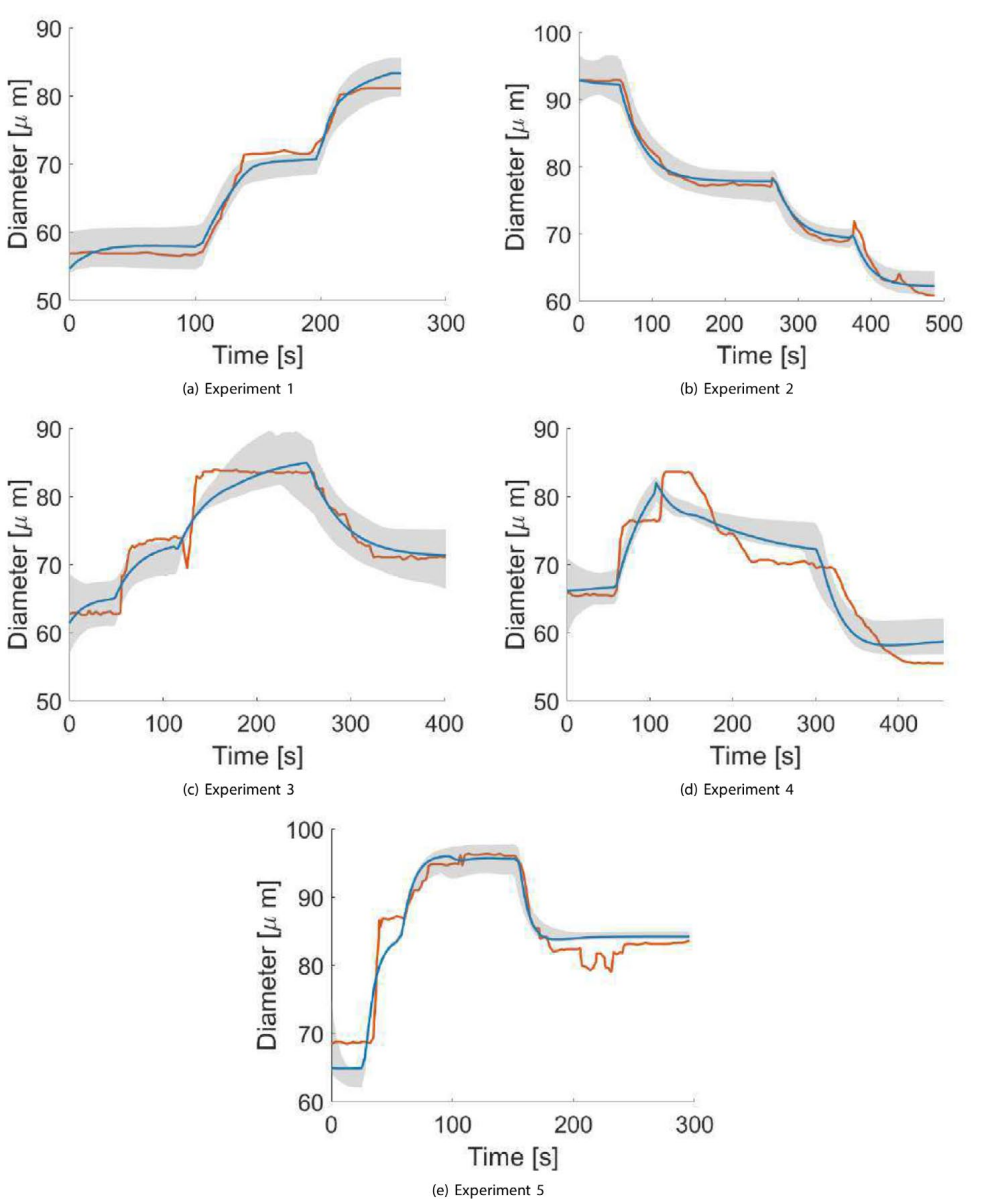
The first‐stage ABC results of the dynamic model simulations when choosing a realization from the final posterior variable space distribution for each experiment, compared to the experimental measurements obtained by Kuo et al. [12, 13] The orange line indicates interpolated experimental measurements, the blue line an example of a simulation trace, and the grey shaded areas depict the uncertainty bounds derived from the posterior distributions, showing the range between the minimum and maximum credible diameters at each time point.

**FIGURE 6:**
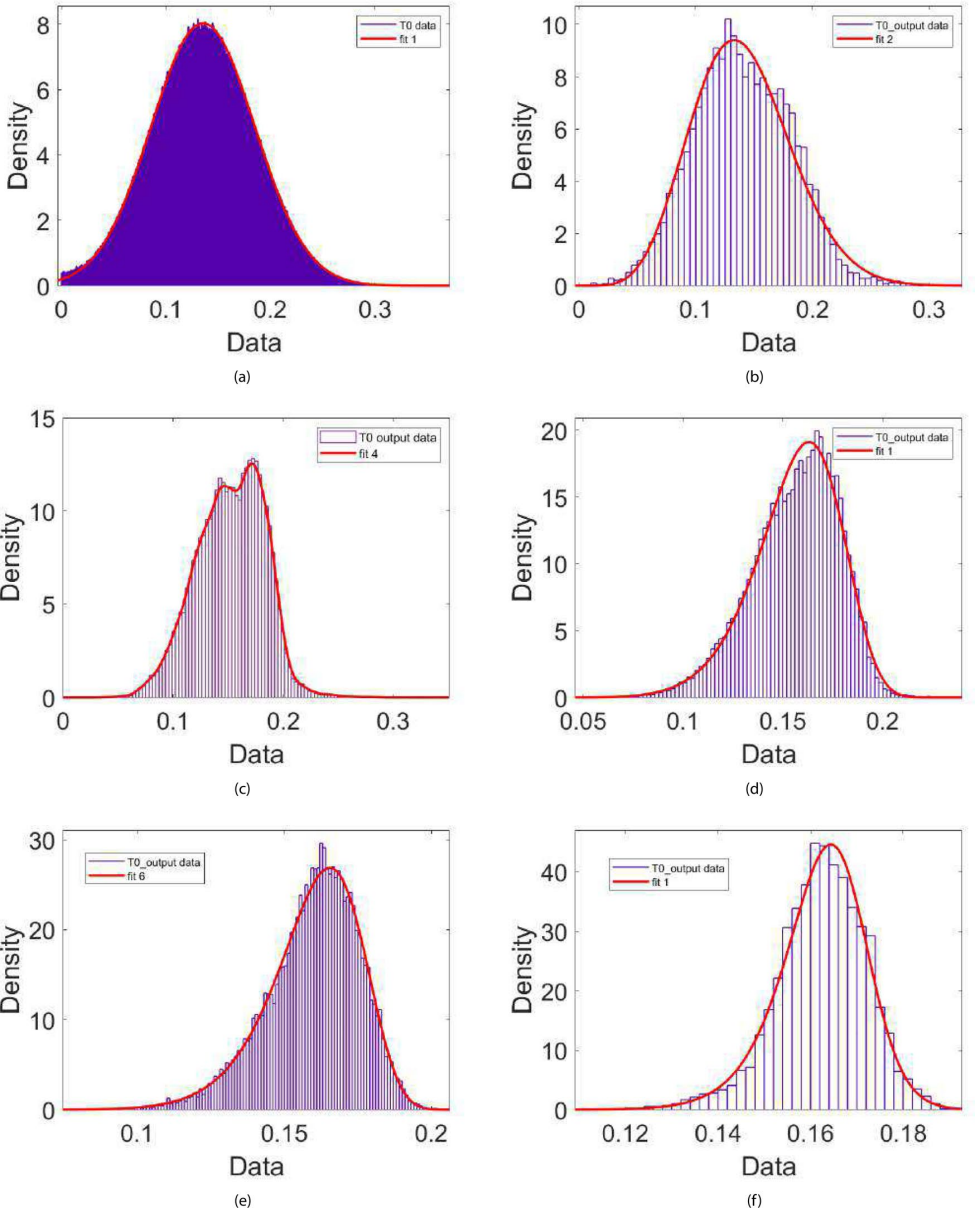
The evolution of the probability distribution function across the five iterations of the first‐stage ABC scheme for the tension reference parameter $T_{0}$ (Pa.m) for experiment 3. The red lines correspond to the univariate distributions (or kernel functions) that are fitted to the posterior data after each iteration. The distributions fitted to the posterior data across the iterations are as follows: (a) normal (*r* = 0), (b) Nakagami (*r* = 1), (c) normal kernel smoothing function (*r* = 2), (d) Weibull (*r* = 3), (e) Weibull (*r* = 4), and (f) Burr (*r* = 5).

Figure 5 demonstrates a comparison between realizations of the measured internal diameter and the values obtained from the dynamic model simulation for each experiment when using a parameter realization obtained from the final posterior distribution. We can see that there is a very good agreement between the simulated and measured diameters. Figure 6 demonstrates an example of the evolution of the posterior distribution function for one of the parameters as it converges towards its final posterior distribution across the first‐stage ABC scheme iterations. We can see that the range (and hence the uncertainty) in the parameter values narrows, and the distribution changes from a normal to a slightly left‐side skewed (Weibull) distribution as it converges across the iterations. The acceptance rate (μ±α) across the five examples for the first‐stage ABC scheme for each iteration were as follows: $\left(1.47\%\pm 0.41\%\right),\left(0.86\%\pm 0.35\%\right),\left(0.89\%\pm 0.4\%\right),\left(0.75\%\pm 0.21\%\right),\text{and}\left(0.58\%\pm 0.29\%\right)$.

**FIGURE 7:**
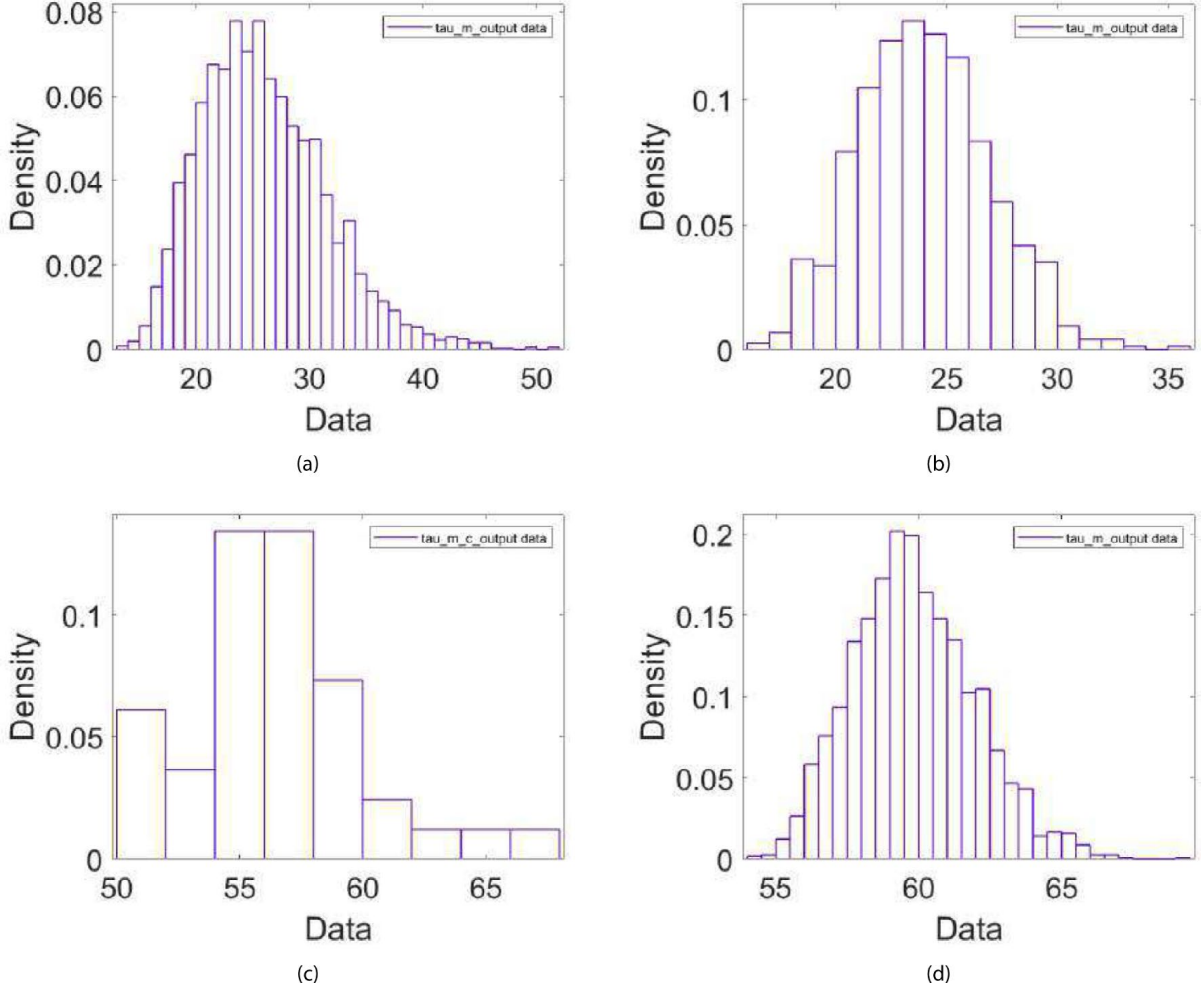
The final first‐stage posterior distributions of $\tau_{m}$ (in seconds) from experiments (3 and 5) involving myogenic‐induced dilation due to a decrease in wall tension (top figures (a) and (b)), and from experiments (2 and 4) involving myogenic‐induced constriction due to an increase in wall tension (bottom figures (c) and (d)).

Figure 7a,b demonstrate the final posterior distribution for $\tau_{m}$ for experiments involving myogenic‐induced dilation (due to a decrease in wall tension), while Figure 7c,d demonstrate the distribution of $\tau_{m}$ obtained from experiments involving myogenic‐induced constriction (due to an increase in wall tension). While there is a general agreement in the final distributions of $\tau_{m}$ within each group (row), we can see that $\tau_{m}$ in the case of a constriction response converges to a final distribution encompassing greater values compared to that of myogenic‐induced dilation. We can approximate the time constants for myogenic dilation and constriction directly from the experimental measurements, by calculating the time it takes from the initiation of the myogenic response for the vessel diameter to drop to 1−1/e of its value. Indeed, the interquartile values of $\tau_{m}$ for myogenic‐induced constriction of 48–61 s are greater than those calculated for myogenic‐induced dilation of 21–34 s. A similar trend can be noticed for the extracted variable distribution of $S_{m}$ (results not shown), in which the distribution tends to be the same within the experiments involving myogenic‐induced constriction or those involving myogenic‐induced dilation, but the distribution in the case of dilation encompass values that are higher (≈3−5 interquartile values) compared to the distribution in the case of constriction (≈1−3 interquartile values).

This effectively demonstrates how sequential ABC schemes can not only be used for model parameter calibration and validation but can also give insight into the mechanistic features of the dynamical system that the model is trying to simulate; in this case, it elucidates the differences in the myogenic time response and sensitivity between vessel dilation and constriction. Accordingly, for the second‐state ABC in which we seek a unified parameter distribution based on all experiments simultaneously, we differentiated between the two cases of myogenic‐induced constriction and dilation, as described in Section 2.5.

**FIGURE 8:**
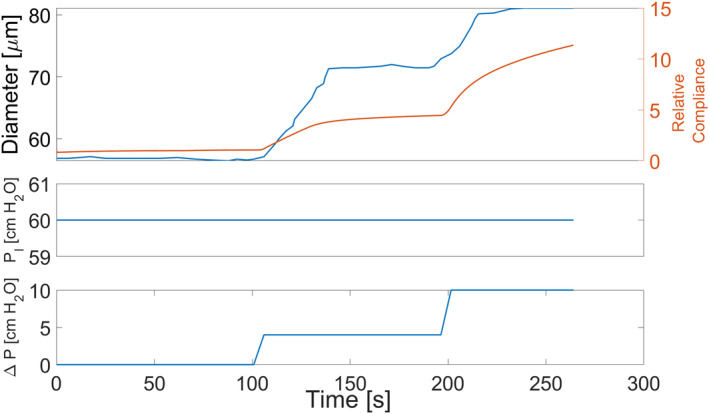
The changes in the internal diameter of the in silico vessel alongside the corresponding variations in vessel compliance (relative to baseline), according to the simulation outputs when running the ODE numerical model with the conditions specified for experiment 1.

Finally, one key benefit of the in silico model is its ability to generate additional relevant metrics, such as vessel wall compliance, and to illustrate how these metrics evolve over time in response to physiological changes in ΔP and $P_{I}$. These data can then be compared to the measured (diameter) and the controlled (ΔP and $P_{I}$) variables to assess and validate the relationship between them. Figure 8 shows an example from a simulation conducted during experiment 1, highlighting the variations in vessel compliance (relative to baseline) and internal diameter in response to changes in ΔP.

### 3.3 Results of the Second‐Stage ABC

**FIGURE 9:**
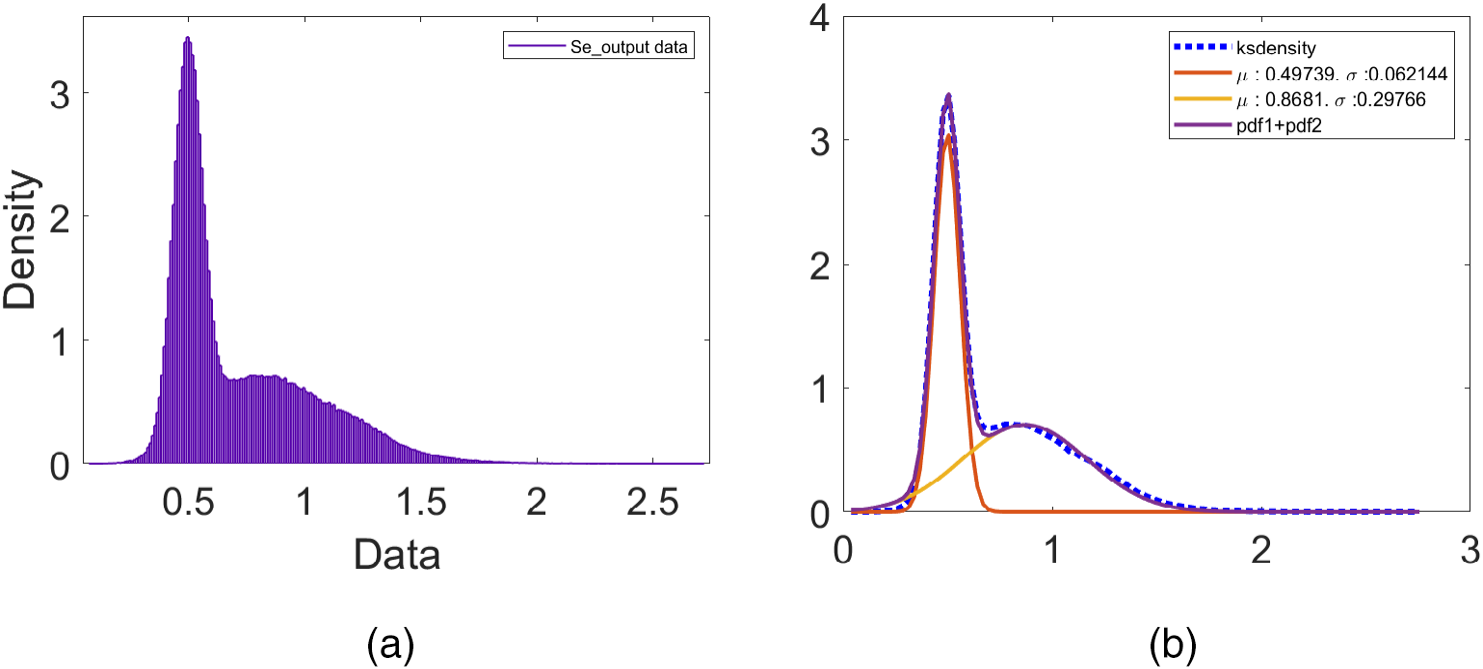
(a) The posterior bimodal distribution for the parameter $S_{e}$, obtained after the first iteration of the second‐stage ABC scheme for group B experiments (experiments 1, 2, and 4). (b) The prior bimodal distribution (purple), constructed from the weighted mixture of two normal distributions (orange and red) to fit the posterior data (blue).

Note that after the first iteration in the second stage ABC scheme, before the parameter space starts to converge, the posterior parameter distributions can (and tend to be) be multimodal (mostly bimodal), and hence cannot be fit by the univariate distribution options offered by the MATLAB DistributionFitter toolbox. In that case, if upon visual inspection the distribution is found to be bimodal, then a separate algorithm, developed to fit a bimodal distribution to the data through the weighted mixture of two normal distributions, is used. Figure 9a shows the initial bimodal distribution for the $S_{e}$ parameter after the first iteration of the second‐stage ABC algorithm for experiments group B, and Figure 9b demonstrates how the distribution can be approximated from the weighted mixture of two normal distributions. The multimodal parameter distribution would then gradually converge into a more pronounced univariate distribution across the iterations, as shown for the final distribution in Figure 11b.

**FIGURE 10:**
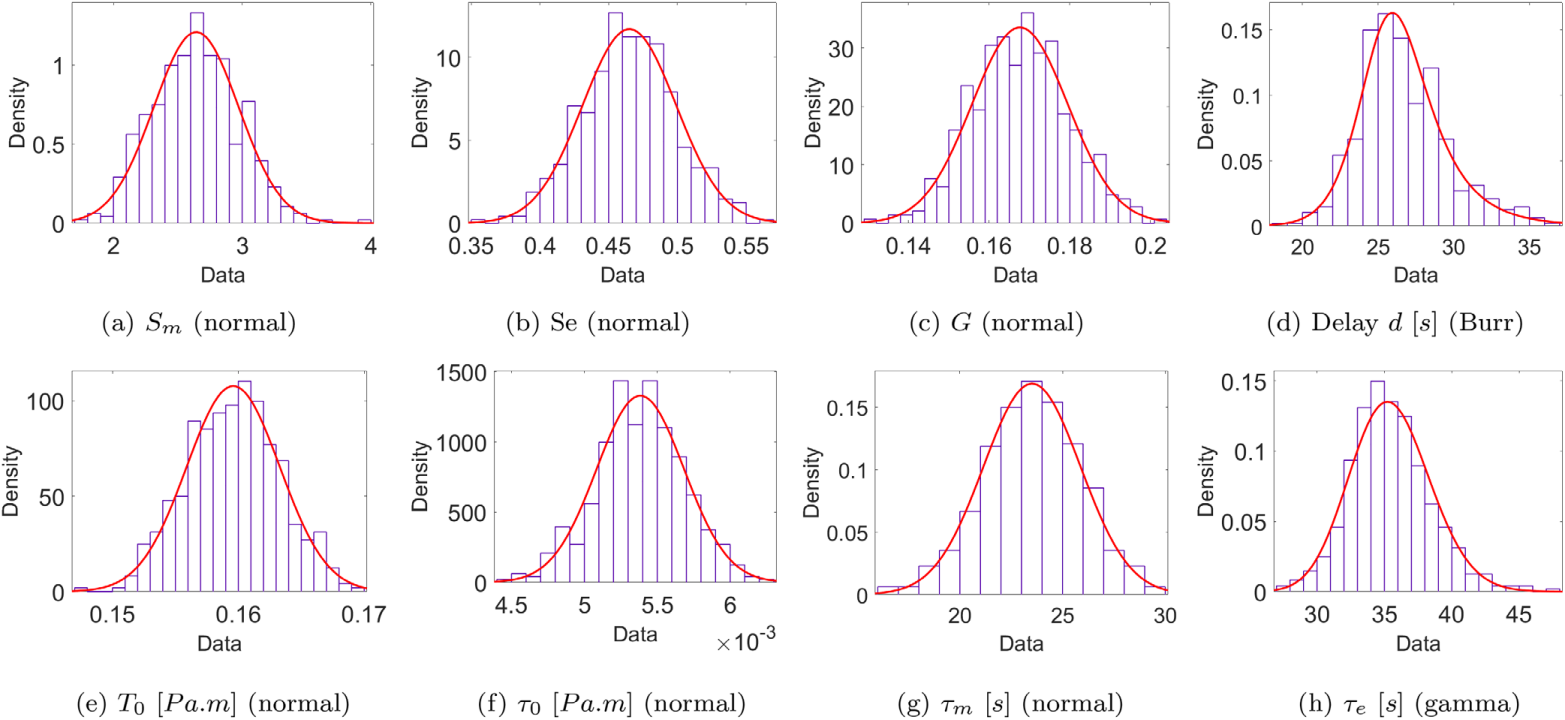
The final posterior distribution of the eight model parameters obtained from the second‐stage ABC scheme (5 iterations) for group A experiments (1, 3, and 5), in which the myogenic response (if any) results in dilation. The best‐fit univariate distribution (red curves) family type is indicated below each figure.

**FIGURE 11:**
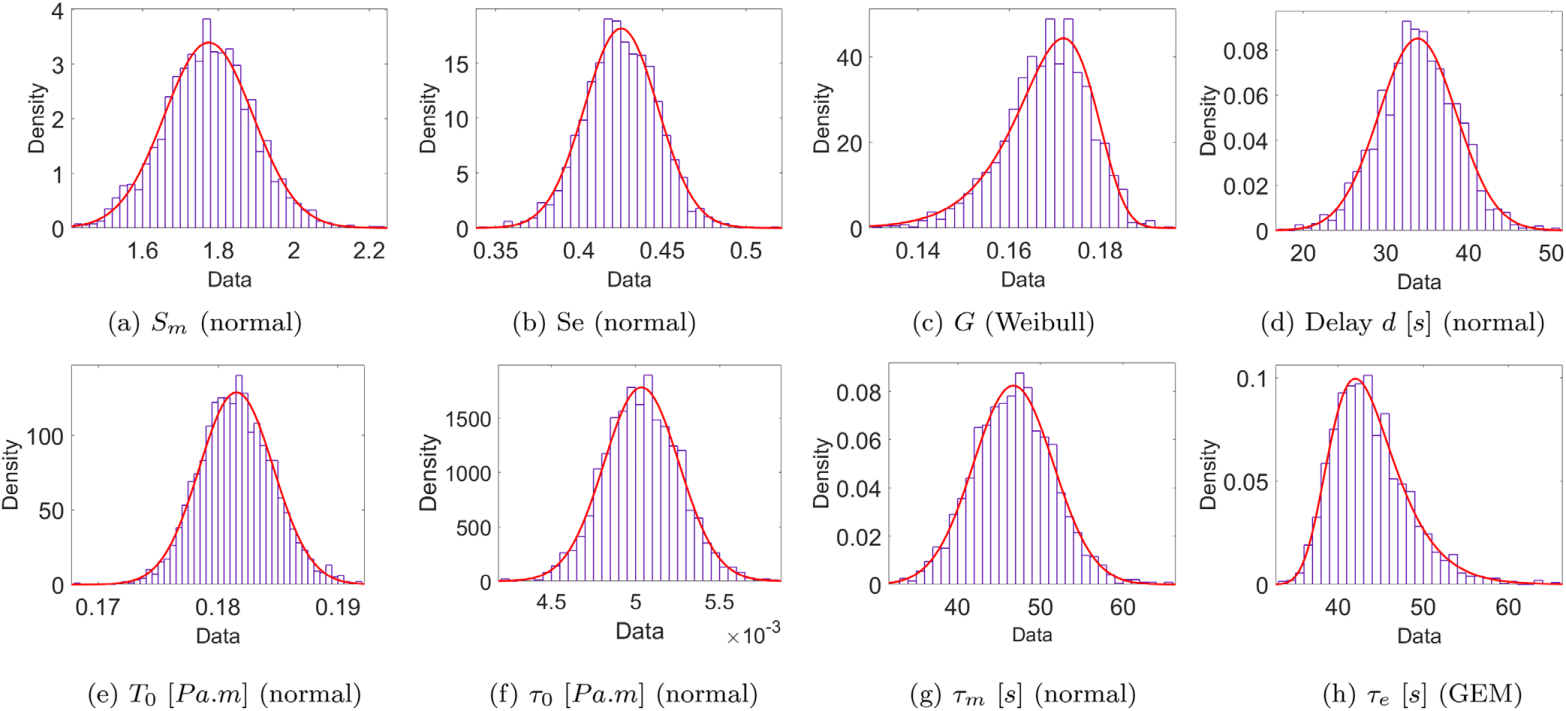
The final posterior distribution of the eight model parameters obtained from the second‐stage ABC scheme (5 iterations) for group B experiments (1, 2, and 4), in which the myogenic response (if any) results in constriction. The best‐fit univariate distribution (red curves) family type is indicated below each figure (GEM = generalized extreme value).

**FIGURE 12:**
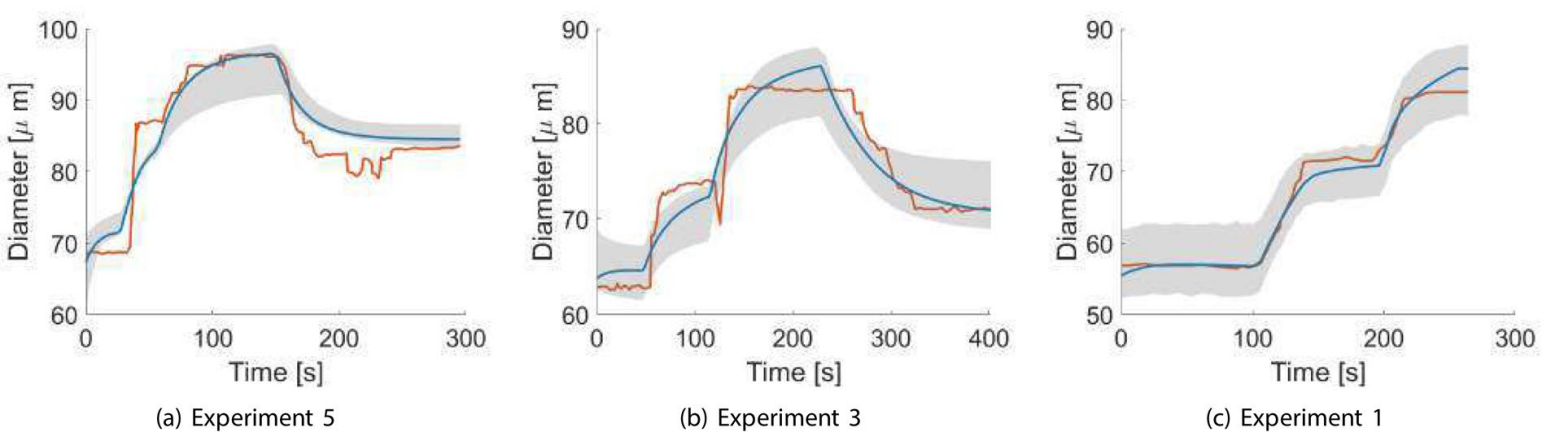
Examples comparing the traces of the simulated datasets to those of experimental measurements for each of the experiments in Group A, after sampling from the unified parameter distribution in Figure 10 obtained from the second‐stage ABC scheme. The orange line indicates interpolated experimental measurements, the blue line is an example of a simulation trace, and the grey shaded area depicts the uncertainty bounds derived from the posterior distribution, showing the range between the minimum and maximum credible diameters at each time point.

**FIGURE 13:**
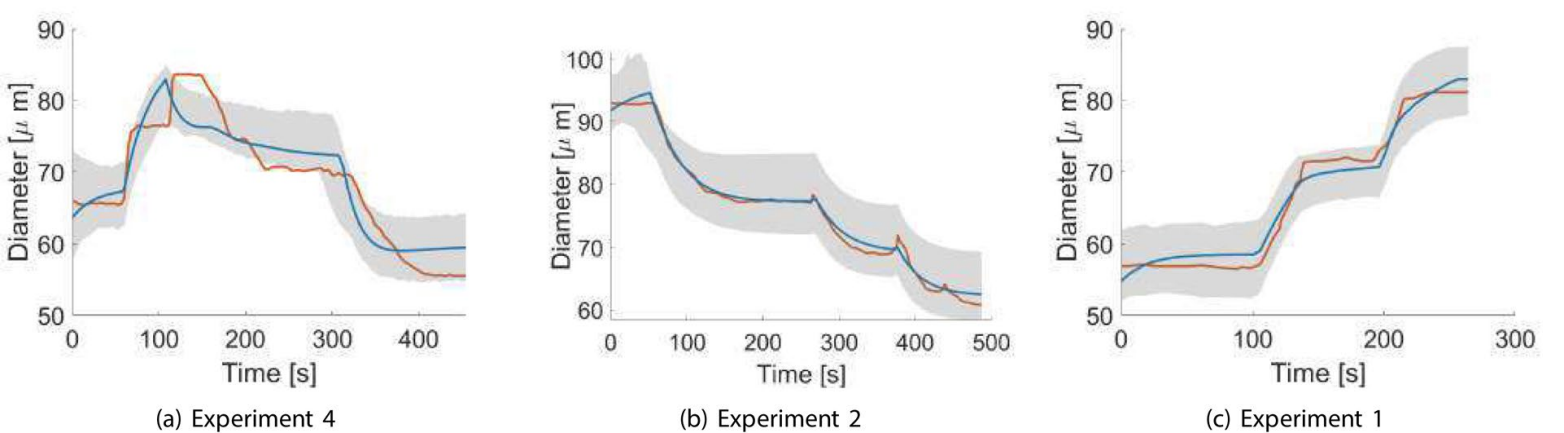
Examples comparing the traces of the simulated datasets to those of experimental measurements for each of the experiments in Group B, after sampling from the unified parameter distribution in Figure 11 obtained from the second‐stage ABC scheme. The orange line indicates interpolated experimental measurements, the blue line is an example of a simulation trace, and the grey shaded areas depict the uncertainty bounds derived from the posterior distributions, showing the range between the minimum and maximum credible diameters at each time point.

Figures 10 and 11 show the final unified model parameter distributions for group A (experiments 1, 3, and 5 where the myogenic response elicits dilation) and group B (experiments 1, 2 and 4, where the myogenic response is constriction‐based), after 5 iterations (*r* = 5) of the second‐stage ABC algorithm. Figures 12 and 13 illustrate comparisons between the measured internal diameter and the values obtained from the dynamic model simulation for each experiment when using a parameter realization obtained from the final posterior distribution. The acceptance rate (μ±α) across the two groups for the second‐stage ABC scheme for each iteration were as follows: 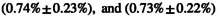.

## 4 Discussion

In this study, we have presented a two‐stage semi‐automated parameter calibration algorithm and used it to calibrate parameter values for the vessel compliance model, which incorporates the myogenic and endothelial responses. The experimental data used for the calibration consist of diameter traces of individual arteriolar vessels in response to the stimulation of the myogenic or endothelial mechanisms, as well as their interactions. Five out of the six experiments available across the two studies by the same research group [12, 13], were ultimately used for parameter calibration.

The five experiments were pooled from two studies [12, 13] by the same group, utilizing identical techniques for microvessel extraction, preparation, and cannulation, as well as the same experimental apparatus and setup for manipulating the controlled variables and measuring the diameter traces. Furthermore, extracted mirovessels are of the same type (porcine coronary arterioles), thereby eliminating any potential confounding factors arising from differences in the organism [14] or the organ [38] that would give rise to discrepancies in the autoregulatory response. Finally, all the microvessels pooled across the two studies share similar characteristic features (in vivo diameters in the range of 40−80μm, and lengths in the range of 0.7−1.1mm), which makes it likely that they were sourced from the same anatomical region and share comparable generation numbers, and thus reduces the chance of different response dynamics due to spatial heterogeneity (i.e., different locations) along the vessel network. In light of all of this, the microvessels across the five experiments likely share similar response dynamics, which justifies the effort to generate a single parameter distribution from all of the experiments simultaneously. A single parameter distribution that is representative of all the experiments and their control conditions, while providing a satisfactory fit for all the experimental measurements, provides strong grounds for the practical identifiability and future predictive capacity of the model and minimizes the likelihood that any good fit could be the result of over‐fitting to particular experimental conditions. The close alignment in Figures 12 and 13 between the experimental measurements across the five experiments and the model simulation output whose parameters are generated from a unified distribution, combined with the fact that all of the model parameters are globally identifiable according to the results of the structural identifiability analysis in Section 3.1, provide robust evidence that the parameter estimation process is reliable, and strongly supports the predictive capability of such models for future clinical applications.

In the second‐stage ABC scheme, and based on the results of the first‐stage scheme, the experiments were divided into two groups (with one experiment present in both groups), to distinguish between myogenic‐induced dilation and constriction. The distributions of the parameters characterizing the myogenic response ($S_{m},\tau_{m},\text{and}\;T_{0}$) are different between the two groups, with the constriction‐based myogenic response exhibiting lower values of $S_{m}$, higher values of $\tau_{m}$, and slightly higher values of $T_{0}$. Hence, it appears that the vessel responds faster and stronger to a drop in blood pressure via myogenic‐induced dilation than to an increase in blood pressure eliciting myogenic‐induced constriction. The mechanisms underlying this greater reactivity to hypotension than to hypertension may be related to a greater influence of vasodilator than vasoconstriction mediators on intracranial vascular tone [39, 40]. In other words, ensuring proper oxygenation might be more important than exerting protective influences elicited by hypertension. It is worth noting that other autoregulatory models have not differentiated between myogenic‐induced dilation and constriction, often using the same time constant for both. The differences in the parameter distributions characterizing the myogenic response illustrate how adopting a suitable parameter calibration scheme to analyze experimental data could potentially provide valuable insights into the mechanisms and pathways of dynamical biological systems under study. Meanwhile, there is a very good overall agreement and overlap in the remaining model parameter values between groups A and B, demonstrating general consistency and reliability of the compliance feedback model to reproduce the vessel response dynamics.

For the second‐stage ABC scheme, the final parameter space distribution within each group was collectively calibrated based on data from all three experiments in that group; thus, it is understood as somewhat of a compromise between the optimal distributions of the three experiments within each group. This explains why the agreement between the simulated and measured traces in Figures 10 and 11 is slightly less pronounced than in the plots in Figure 5 from the first‐stage ABC scheme, in which the parameter distributions were independently calibrated for each experiment. This is evidenced by the lower and statistically different (p<0.05) final error tolerances (mean ± std) observed in the first‐stage scheme compared to the second‐stage scheme across the error norms (in μm): $L_{2}$ (2.72±0.078 vs. 3.85±1.45), $L_{4}$ (0.115±0.08 vs. 0.238±0.156), and $L_{\infty }$ (4.165±0.804 vs. 7.541±0.827). Furthermore, the parameter posterior distributions from the second‐stage scheme exhibit a broader range, resulting in greater uncertainty in the simulation outputs, as reflected in the wider gray uncertainty bounds surrounding the second‐stage results. This outcome is anticipated, given that the second‐stage posterior distribution for each of group A and B incorporates the experimental setup and outcomes from all three experiments simultaneously. Nonetheless, sampling from the final unified distribution has resulted in an overall very good agreement among all five experiments in groups A and B, meaning that the compliance feedback dynamic model can achieve great accuracy following parameter calibration, all while maintaining a very low computational cost: the simulation duration per realization for a single vessel was <3s when run on a Desktop computer, corresponding to a real‐life duration of 300s. Such a computationally affordable model becomes a particularly attractive option when scaling and modelling the behavior of multiple interconnected vessels within a vascular network, such as in the network and multiscale models by Daher and Payne [2, 3, 4]. Furthermore, while the final distributions in Figures 10 and 11 are meant to accommodate data from all three experimental setups within each group, the distributions are sufficiently narrow that a meaningful range of the parameter values can be obtained and used in future simulations.

As discussed in Section 3.2, one significant advantage of in silico models is their capacity to produce additional relevant metrics, such as vessel wall compliance, and to demonstrate how these metrics change over time in response to physiological variations in ΔP and $P_{I}$. Figure 8 illustrates that the variations in vessel compliance can be compared with these variables to evaluate and confirm their interrelationship, as well as to determine whether the model operates as anticipated. In the figure, the changes in vessel compliance align with the alterations in vessel diameter and ΔP, which is consistent with expectations, since the autoregulatory responses act in response to physiological perturbations by changing the vessel wall compliance, which in turn changes the pressure‐radius relationship of the vessel and gives rise to changes in the diameter. In a physiological context, changes in the diameter influence fluid flow resistance, facilitating the restoration of normal flow levels. Initially, the compliance is set to its baseline levels. Given that the value for $\Delta C^{\pm }/C_{b}$ is fixed to 10, the maximum relative compliance achievable in the simulations is 11. As depicted in Figure 8, with a ΔP of 10 cm H₂O (7.36 mmHg), the relative compliance—and thus the stiffness of the vessel—approaches its saturation point according to the model. This suggests that any further increase in ΔP or decrease in $P_{I}$ will not significantly impact vessel compliance or the pressure‐radius relationship. Consequently, this behavior mimics the nonlinear stiffness and elastic properties characteristic of blood vessels [41, 42].

We note that experimental data can be affected by confounding factors and corrupted by artefacts and noise, in addition to errors in the interpolation process, such that an accurate calibration can sometimes be difficult to achieve. Data thus have always to be treated with caution. Furthermore, we recognize that the compliance feedback model, while physiologically grounded, is a simplified representation of the vascular smooth muscle cell dynamics. We also note that we have treated the maximal and minimal fractional change in compliance parameters $\left(\frac{\Delta C^{\pm }}{C_{b}}\right)$, as well as the baseline compliance, $C_{b}$ as fixed. The values for $\left(\frac{\Delta C^{\pm }}{C_{b}}\right)$ were set to 10 and 0.8, respectively, based on the values from a previous compliance feedback model [34], and the value of $C_{b}$ was determined from the independent ring model [27] and fixed, since previous parameter sensitivity analyses of similar models [2, 3] revealed relatively little influence of these parameters. Incorporating those additional parameters into the variable space could have resulted in a better fit between the simulated data set and the experimental measurements, but we opted not to include them to maintain a reasonable parameter space dimensionality, with eight model parameters. In addition, potentially better fits between the simulated datasets and the measurements, as well as narrower final posterior distributions, could have been obtained by increasing the number of iterations in the second‐stage ABC scheme, but we have generally found that after conducting five iterations the decrease in the error norms and the changes in the parameter distributions are very insignificant, especially when contrasted against the increased computational cost. We note that in the future, the semi‐automated algorithm could be potentially streamlined so that it is entirely automated, by exclusively using smoothing kernels, as opposed to relying on best‐fitting with known univariate (or bivariate) distributions, to fit the posterior distributions. However, the designation of the error tolerances, which get gradually reduced in the SMC scheme across the iterations, was heuristic in the sense that it was based on the error tolerances of the accepted realizations from the previous iteration and thus necessitated intervention in between the ABC iterations.

As discussed in Section 1.3, ABC schemes for parameter calibration can also address uncertainties in model simulations, a critical capability in cases where measurement errors and noise are poorly characterized, and where uncertainties need to be propagated in time, such as in the case of the experimental studies by Kuo et al. [12, 13] utilized in this study. The uncertainty bounds are represented by the grey‐shaded regions in Figures 5, 12, and 13, which compare simulated and measured diameter traces. From a Bayesian perspective, simulation outcomes can be treated as probabilistic, assigning probability values to potential outcomes for uncertainty quantification (though not performed here), given that each parameter value corresponds to a specific probability density function. Moreover, the initial conditions of the state variables ($x_{i}={\left[x_{\mathrm{ei}},x_{\mathrm{mi}},R_{i}\right]}^{T}$) were treated as uncertain parameters, enabling their values and distributions to adapt dynamically during the SMC ABC calibration process as the error norms were minimized. As a result, the steady‐state conditions that informed the prior distributions in Section 2.4.2 were occasionally no longer valid, as observed in certain diameter traces shown in Figures 5, 12, and 13. To ensure that simulations consistently begin from steady‐state conditions, one could, in principle, filter the accepted realizations or use them to recalibrate steady‐state values iteratively at each stage of the SMC ABC process, as outlined in Section 2.4.2. However, given the absence of experimental error information from the original studies, we opted to forgo this step, instead allowing the algorithm to autonomously determine optimal initial conditions without imposing additional constraints.

## 5 Conclusion

In summary, we have demonstrated that the chosen functional form of the compliance model provides a concise yet accurate representation of vessel mechanics, particularly in regard to the myogenic and endothelial autoregulatory responses of arteriolar vessels. While hemodynamic models traditionally lean towards either law‐driven or data‐driven formulations, the calibration of the parameter space and the functional form of the compliance feedback model ensure a balanced integration of both approaches. The model's mathematical simplicity, computational efficiency, physiological relevance, and strong agreement with experimental data make it a robust candidate for inclusion in hemodynamic simulations, which in turn can help bridge the gap between mathematical models and clinical studies and increase the translatability of such mathematical models into the clinical environment.

## Ethics Statement

The author has nothing to report.

## Conflicts of Interest

The author declares no conflicts of interest.

## Data Availability

The data that support the findings of this study are available from the corresponding author upon reasonable request.
